# Transcriptome analysis reveals plasticity in gene regulation due to environmental cues in *Primula sikkimensis*, a high altitude plant species

**DOI:** 10.1186/s12864-019-6354-1

**Published:** 2019-12-17

**Authors:** Priya Darshini Gurung, Atul Kumar Upadhyay, Pardeep Kumar Bhardwaj, Ramanathan Sowdhamini, Uma Ramakrishnan

**Affiliations:** 1 National Center for Biological Sciences (NCBS), Tata Institute of Fundamental Research, GKVK Campus, Bellary Road, Bengaluru, Karnataka 560065 India; 2 Manipal University, Manipal, India; 3 Present Address: Thapar Institute of Engineering & Technology, Department of Biotechnology, Patiala, Punjab 147004 India; 4 Institute of Bioresource & Sustainable Development, A National Institute under Department of Biotechnology, Ministry of Science & Technology, Government of India, Gangtok, Sikkim 737102 India; 5 Present address: Institute of Bioresources and Sustainable Development, Meghalaya, 6th Mile, Upper Shillong, Meghalaya 793009 India

**Keywords:** Gene expression, Transplant experiment, Transcriptomics, Climate change, Range limits

## Abstract

**Background:**

Studying plasticity in gene expression in natural systems is crucial, for predicting and managing the effects of climate change on plant species. To understand the contribution of gene expression level variations to abiotic stress compensation in a Himalaya plant (*Primula sikkimensis*), we carried out a transplant experiment within (Ambient), and beyond (Below Ambient and Above Ambient) the altitudinal range limit of species. We sequenced nine transcriptomes (three each from each altitudinal range condition) using Illumina sequencing technology. We compared the fitness variation of transplants among three transplant conditions.

**Results:**

A large number of significantly differentially expressed genes (DEGs) between below ambient versus ambient (109) and above ambient versus ambient (85) were identified. Transcripts involved in plant growth and development were mostly up-regulated in below ambient conditions. Transcripts involved in signalling, defence, and membrane transport were mostly up-regulated in above ambient condition. Pathway analysis revealed that most of the genes involved in metabolic processes, secondary metabolism, and flavonoid biosynthesis were differentially expressed in below ambient conditions, whereas most of the genes involved in photosynthesis and plant hormone signalling were differentially expressed in above ambient conditions. In addition, we observed higher reproductive fitness in transplant individuals at below ambient condition compared to above ambient conditions; contrary to what we expect from the cold adaptive *P. sikkimensis* plants.

**Conclusions:**

We reveal *P. sikkimensis’s* capacity for rapid adaptation to climate change through transcriptome variation, which may facilitate the phenotypic plasticity observed in morphological and life history traits. The genes and pathways identified provide a genetic resource for understanding the temperature stress (both the hot and cold stress) tolerance mechanism of *P. sikkimensis* in their natural environment.

## Background

Understanding constraints on species’ range limits have long been a primary goal of ecologists [1]. Climate has been recognized as a factor controlling species’ range limit [2]. When the climate changes gradually, ecosystems and species can evolve together. However, given the current rate at which the climate is changing [3], concerns are rising about the capacity of species to adapt. Sessile organisms such as plants have to be considerably more adaptable to stressful environments and must acquire greater tolerance to multiple stresses than animals. It is well known that environment induced phenotypic plasticity plays an important role in adaptation [4, 5], and plant phenotypic responses to altered environmental stresses are mainly regulated through gene expression [6, 7]. Thus, understanding plasticity in gene expression in natural systems is crucial, for predicting and managing the effects of climate change on plant species.

Variation in gene expression patterns plays a key role in the evolution of phenotypes [8] that allow an organism to acclimatize to stress [9, 10]. For example, thermal stress is considered a major constraint to plant reproduction. Almost all organisms respond to thermal stress by synthesizing *heat-shock proteins* (*HSPs*) [11–13]. However, different species respond differently to similar stress conditions; cold stress induces over expression of the *C-repeat binding factor* (*CBF*) genes in *Arabidopsis thaliana* [14] and induces over expression (10-fold upregulation) of *OsCYP19–4* gene in *Oryza sativa* [15]. Plants may respond differently to multiple stress conditions [16], and the molecular mechanisms associated with multiple stresses might differ from those related to single stress [17, 18]. While many studies provide insight into plant responses to single stresses under controlled conditions [19–21], responses to changing conditions in the natural environment remains less understood.

Variation in gene expression under different conditions can be identified through genome-wide transcriptome analysis [22] using RNA sequencing (RNA_seq) [6, 23]. Application of RNA-seq to non-model species allows the use of their transcriptomes to understand their responses to changes in the environment [24, 25]. Many studies clearly demonstrated/ suggested that adaptive plasticity can processed through transcriptome variation [26–29], and much work is needed in these regards.

Altitudinal gradients provide a wide temperature range over a very short distance [30] and are therefore ideal to study potentially adaptive phenotypic variation in plants in the wild. Temperature differences along this fine-scale altitudinal gradients across ‘space’ can be used to infer the potential temporal responses of a population to climate change [31]. Many studies on altitudinal gradient to date have focused on species morphological and physiological differences, or the genetic basis of high altitude adaptations, and few studies have examined the contribution of gene expression level variation along altitudinal gradients [32, 26, 28]. *Primula sikkimensis* (genus *Primula* L.) is high altitude specialist plant, and one of the most dominant and widespread species, distributed along the altitudinal gradient of Sikkim Himalaya (27 °C 62’N, 88 °C 63’E) from 3355 m a.s.l. to 4598 m a.s.l. (field survey during 2012–2015, Lachen valley North-Sikkim). Populations sampled at different altitudes display phenotypic differences. Populations from higher altitudes (~ 4500 m a.s.l.) are smaller with delayed maturity and flowering compared to lower altitude populations (~ 3500 m a.s.l.), which are taller and flower earlier in the spring [33].

In this study we carried out a transplant experiments within and beyond the altitudinal range limit of *P. sikkimensis*. The gene expression profiles of transplant groups were obtained with transcriptome sequencing and we identified differentially expressed genes (DEGs) between within and beyond range transplant groups. The overall objective of this study was to facilitate a better understanding of how the gene expression level variation may have contributed to abiotic stress compensation in *Primula sikkimensis*.

## Results

### Illumina paired-end sequencing and de novo assembly of transcriptome

Illumina paired-end sequencing generated approximately 90 million raw reads (2 × 101 base pair). After pre-processing of raw reads, approximately 60 million reads (R1 = 2 × 94 base pair & R2 = 2 × 101 base pair) were left. In the absence of available reference genome for *P. sikkimensis*, we de novo assembled the transcriptome to be used as a reference for read mapping and gene expression profiling (hereafter referred to as the reference transcriptome assembly). We assembled the high-quality processed reads and the best-combined assembly resulted in 67,201 genes, 81,056 transcripts with a mean length of 785.87 bp and average open reading frame (ORF) length of 468.6 bp. The N50 of contigs was 1359 bp, a total size of 63.4 Mb, and a GC content of 38.99%. Similarly, results of separate assemblies in all the three transplant conditions were documented in Table 1. Only 3% (2647) of the transcripts have putative frameshifts which suggests good quality transcriptome data (Accession number: SRP150603). The raw reads generated from Illumina sequencing were deposited at National Centre for Biotechnology Information (NCBI), SRA with accession numberSRP150603.

**Table 1:** The results of separate transcriptome assemblies of *P. sikkimensis* in all three transplant conditions (ambient, below ambient and above ambient), and the reference assembly generated by combining the reads from all three conditions were documented in tabular form

Transcriptome data analysis	Above ambient	Ambient	Below ambient	Combined assembly
Total Number of genes	44,957	48,674	38,423	67,201
N50 (bp)	1371	1386	1405	1359
Total transcripts	53,133	58,644	44,142	81,056
GC percent	39.89	39.61	40.49	38.99
Average contig length (bp)	475	822	854	785.87

### Functional annotation and identification of differentially expressed genes (DEGs)

Functional annotation of *P. sikkimensis* transcriptome assembly was carried out using TRAPID, in which Plaza database was used. Plaza is a collection of transcripts and genomes of plants. Our annotation resulted in 22,332 (27.6%) of transcripts annotated with GO categories and 26,313 (32.5%) of *P. sikkimensis* sequences annotated with known protein domains.

**Fig. 1:**
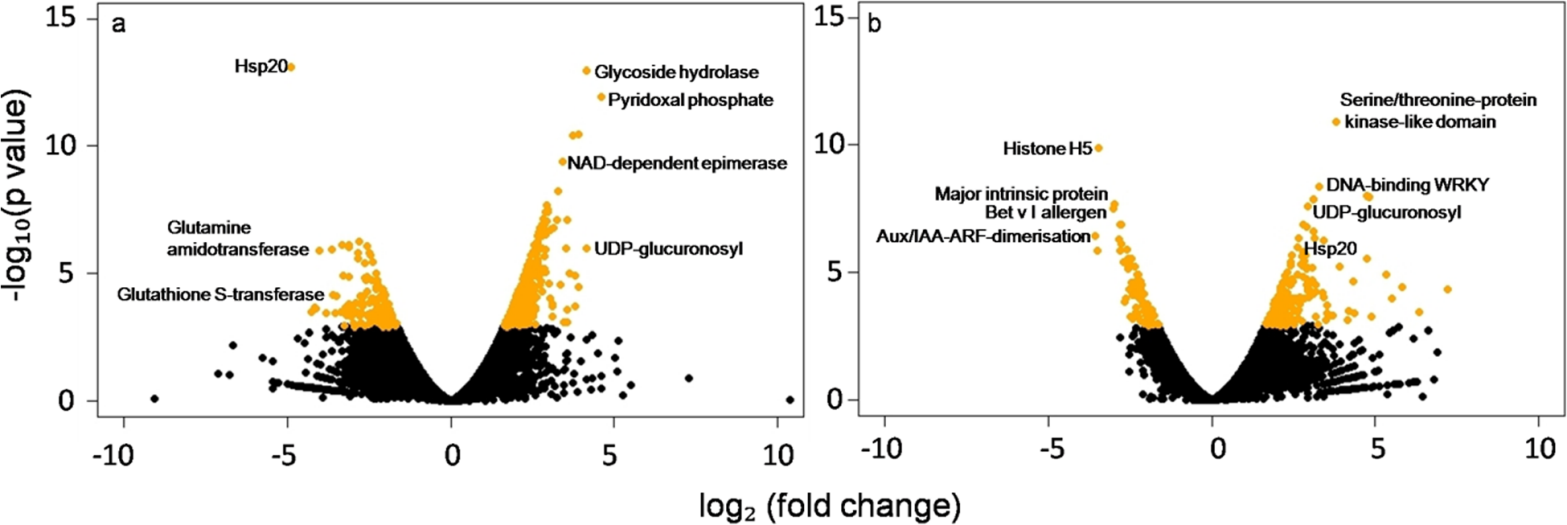
Volcano plots showing differentially expressed genes between (**a**) below ambient vs. ambient and (**b**) above ambient vs. ambient. The y-axis corresponds to the mean expression value of log_10_ (*p*-value), and the x-axis displays the log_2_ fold change value. The orange dots represent the significantly differentially expressed transcripts (*p* < 0.05); the black dots represent the transcripts whose expression levels did not reach statistical significance (*p* > 0.05)

**Fig. 2:**
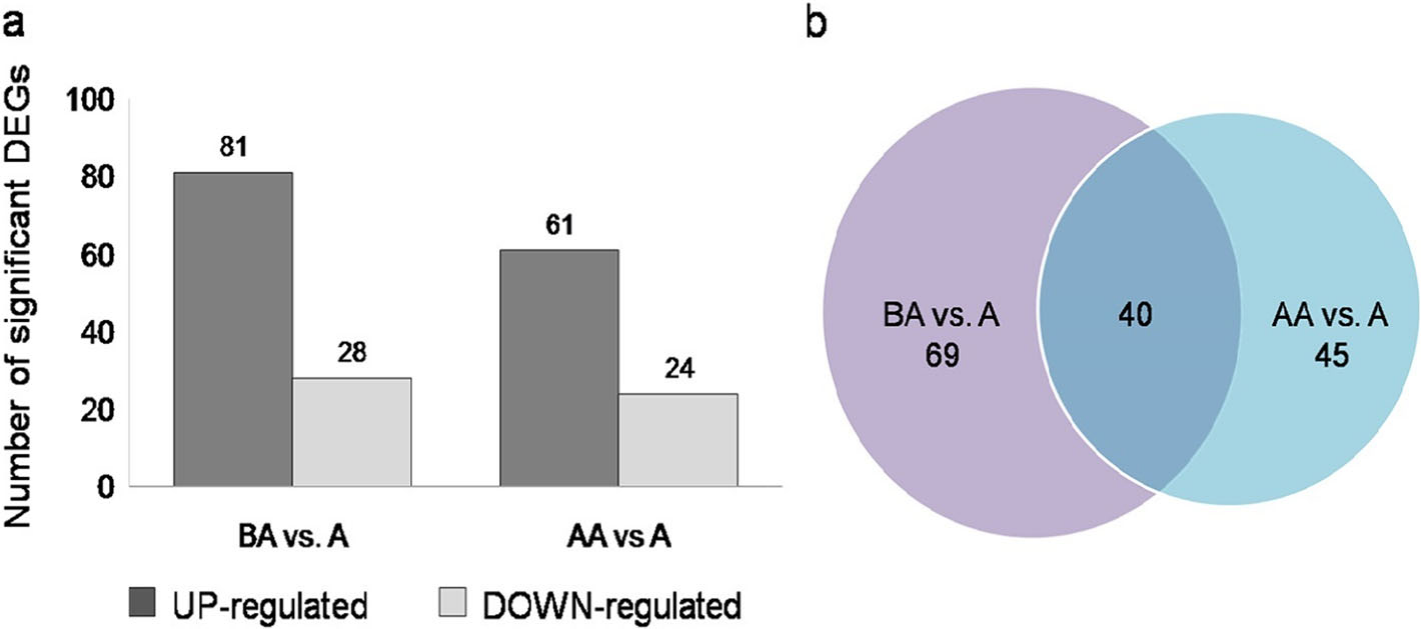
Differential gene expression profiles. **a** A number of up and down regulated genes in the pair-wise comparison between below ambient versus ambient and above ambient versus ambient transplant conditions. **b** Venn diagram presenting the number of unique and overlapping genes between two pair-wise comparisons

Using the RNA-seq data, we derived gene expression profiles in *P. sikkimensis* for all three transplant conditions. We then carried out two comparative transcriptome analyses between Ambient (A) the control, versus Below Ambient (BA), and Above Ambient (AA) transplant conditions. For comparison of differentially expressed genes we used 21,167 transcripts which mapped to the reference transcriptome of *P. sikkimensis*. To judge the significance of gene expression difference from the two pairwise comparisons we identified significantly differentially expressed genes of *P. sikkimensis* as those with log_2_ (fold change) ≥ 2 and log_10_ (*p*-value) < 0.05, as a threshold. A large fold change in expression does not always imply statistical significance, as those fold changes may have been observed in genes that received little sequencing or with many iso-forms [34], therefore we consider both fold change and p-value to identify the significant DEGs. We used volcano plots to show the significant DEGs which relate the observed differences in gene expression to the significance associated with those changes under Cuffdiff’s statistical model (Fig. 1). We found 109 significant DEGs from BA vs. A comparison, 81 up-regulated and 28 down-regulated (Fig. 2a).These genes include *heat shock proteins HSP20, HSP70, Transcriptional factor B3, Methionine synthase, Zinc finger, dTDP-4-dehydrorhamnose reductase, DNA-binding, ATPase,* and *UDP-glucuronosyl* (full list of genes, Additional file 8 Table S3a). From AA vs. A, we found 85 significant DEGs of which 61 were up-regulated and 24 were down-regulated (Fig. 2a). These genes include *Heat shock protein DnaJ, bZIP transcription facto*r and *Histone H5* (full list of genes, Additional file 8 Table S3b). Forty genes were common between the two pair-wise comparisons, whereas 69 and 45 genes were unique to BA vs. A and AA vs. A comparison respectively (Fig. 2b).

### Gene ontology (GO) and pathways mapping of DEGs

DEGs from the two pair-wise comparisons were mapped to GO database and GO terms were assigned. The DEGs had a GO ID and were categorized into small functional groups in three main categories (cellular component, molecular function, and biological process) of GO classification. Based on sequence homology, 42 and 36 functional groups were categorized in BA vs. A, and AA vs. A comparisons, respectively. Among these groups, “cell” and “cell part” were dominant within the “cellular component” category; “binding” and “catalytic” were dominant in the “molecular function” category; and “cellular process” and “metabolic process” were dominant in the “biological process” category (Additional file 4 Figure S4b).

**Fig. 3:**
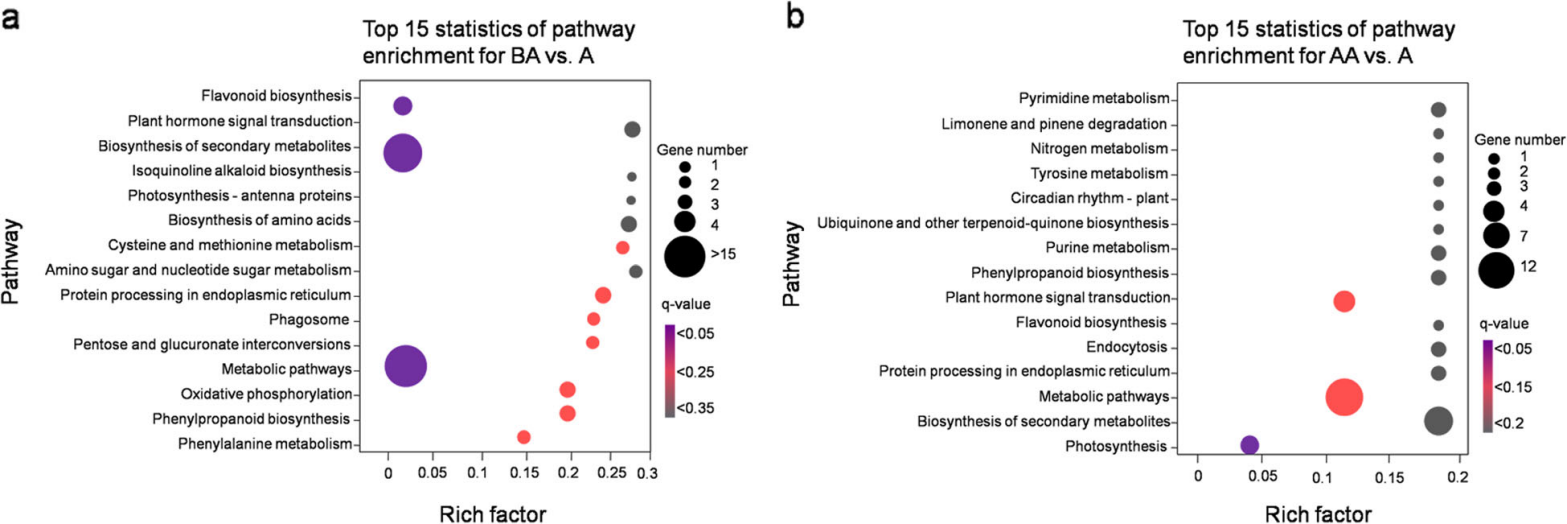
Scatter plot of KEGG pathway enrichment analysis of differentially expressed genes in (**a**) below ambient versus ambient and (**b**) above ambient versus ambient transplant conditions. The number of DEGs in the pathway is indicated by the circle area, and the circle color represents the range of the corrected p-value (q-value) from 0~1. We display the top 15 pathway terms enriched by KEGG database

The biological function associated with significant DEGs were further analyzed in terms of enriched Kyoto Encyclopaedia of Genes and Genomes (KEGG) pathways [35]. The DEGs had a KO ID and were categorized into small pathways. A total of 34 pathways were predicted for BA vs. A comparison and among them, “metabolic pathway”, “biosynthesis of secondary metabolites” and “flavonoid biosynthesis” were the most highly represented categories (Additional file 9 Table S4a). Similarly, 23 pathways were predicted for AA vs. A comparison and among them, “metabolic pathway”, “biosynthesis of secondary metabolites”, “plant hormones signal transduction”, and “photosynthesis” were the most highly represented categories (Additional file 9 Table S4b). The top 15 KEGG pathways of DEGs in these two pairwise comparisons are shown in Fig. 3.

### Validation of RNA-Seq data by real-time quantitative RT-PCR

**Fig. 4:**
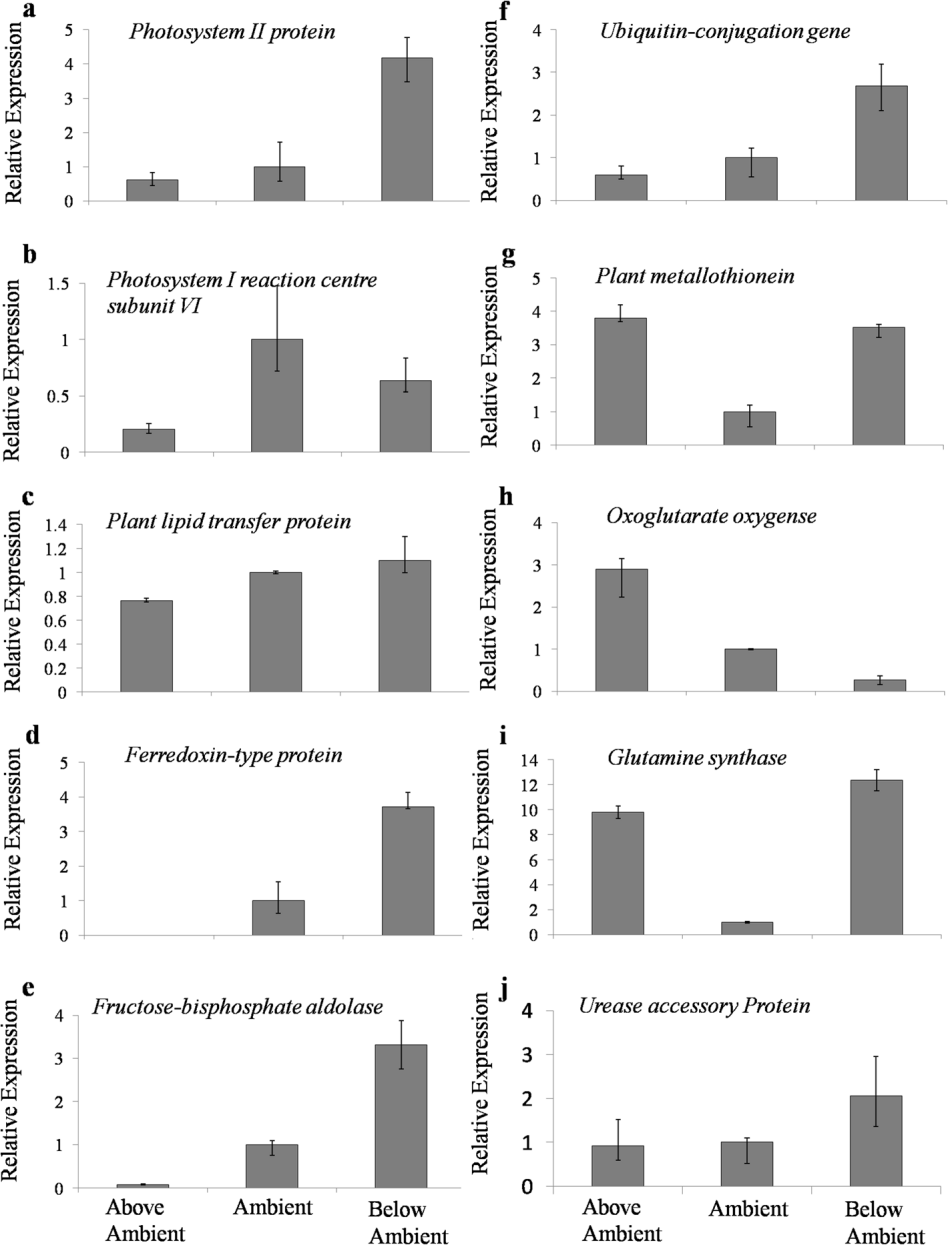
Real-Time PCR analysis of selected genes in AA, A, and BA samples (**a**-**j**). Here the data repersented are realtive quantification (RQ) values of gene expression

To confirm the RNA-Seq data, the transcript level of randomly selected 10 genes was examined by Real-Time quantitative RT-PCR (Fig. 4). All the genes exhibited the same pattern of expression as per FPKM (fragments per kilobase of exon per million fragments mapped) values for A, BA, and AA conditions except for “c15913_g1” annotated as *ferredoxin-type protein*, which was not detected in AA (Fig. 4). Taken together, all the selected genes (Table 2) showed same patterns that were consistent with the RNA-seq data, validating our experimental results.

**Table 2:** List of primers used for Real-Time quantitative RT-PCR

S. No	Gene name	Forward primer (5`-3`)	Reverse primer (5`-3`)
1	*Photosystem II protein PsbR*	AGCTCCCACCTCAAGGAGAT	AGCAACTCTTCAGCCTCTGC
2	*Photosystem I reaction centre subunit VI*	AGGTGGAGGTTGCTGTGACT	CTTCTCTGCGACCGTTAAGC
3	*Plant lipid transfer protein/seed storage*	CAACAGCTGAGAGAACCCATC	GGCAGCTATGCCTTTCATCT
4	*Ferredoxin-type protein*	AAGGAGCTGGTTGTCAAGGA	ATCTGCTCACACATCGCAAG
5	*Fructose-bisphosphate aldolase, class-I*	CCATGATGTGGTGGACGATA	GGCTAGCCTGCGATGTCTAC
6	*Ubiquitin-conjugating enzyme, E2*	AGGCTTCCGTGCTACACAAC	TTAAGGCAGGTTGCTCCTTC
7	*Plant metallothionein, family 15*	GTTAGAACCTGGGTGGCATC	GATCTTTGGCTCGACTTGCT
8	*Oxoglutarate/iron-dependent oxygenase*	CCAGTCAAAGACTCGGAACC	GAAGGAGTCACCGTCTCCAG
9	*Glutamine synthetase/guanido kinase, catalytic domain*	CCCACTTTAGAGCGAGAGACTG	GTGAGATGACGGCGATGAC
10	*Urease accessory protein UreD*	CTCCAAGTTTCCGAGGATTG	CCCTAAGCCAGCACTGTAGC
11	*26 S rRNA*	CCCTGTGGTAACTTTTCTG	GCTCGTTTGATTCTGATTTC

### Differences in fitness-related traits of transplants across three transplant sites

**Fig. 5:**
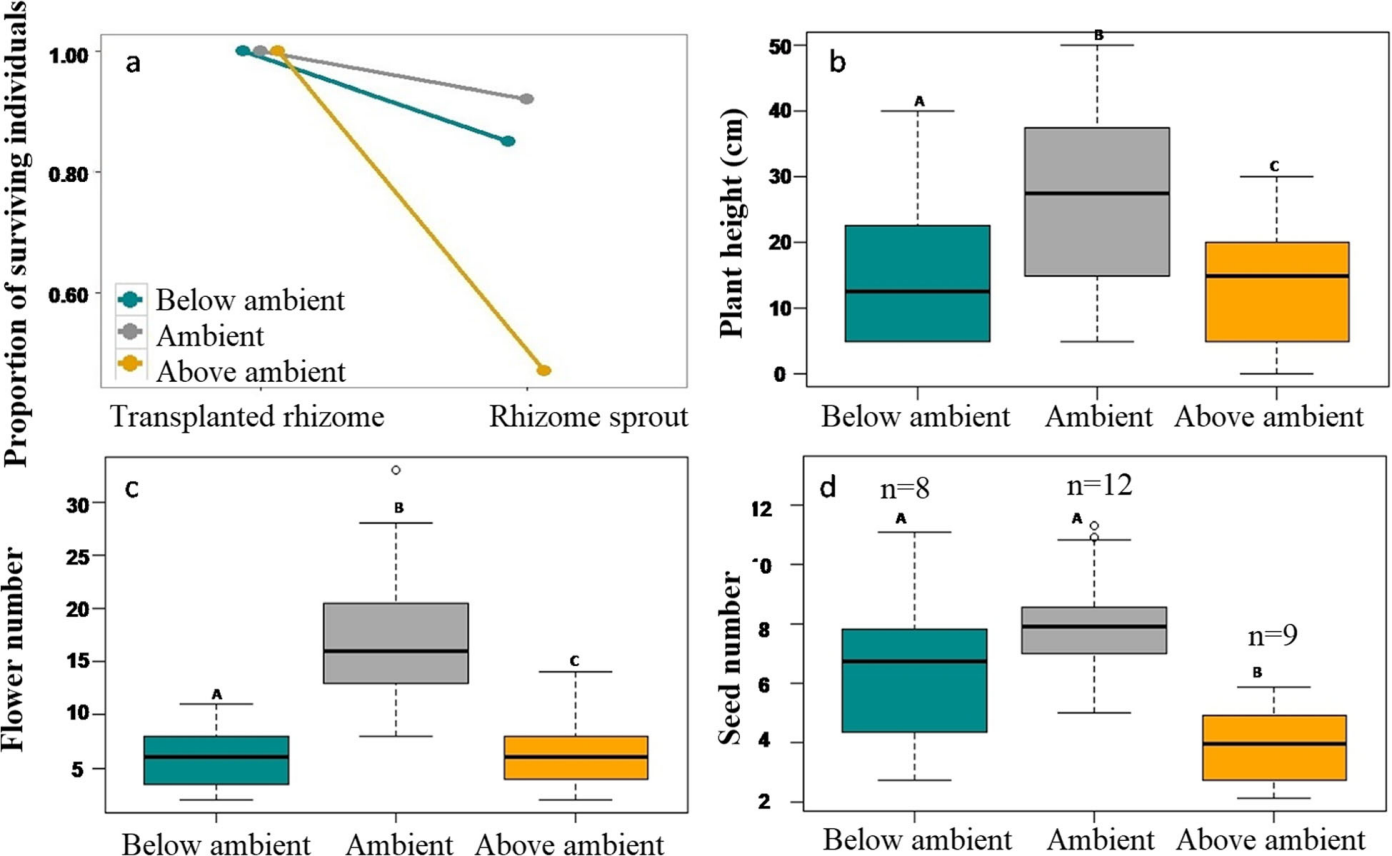
**a** Survival of transplanted rhizomes of *P. sikkimensis* at below ambient, ambient, and above ambient transplant sites. **b** plant height, **c** flower number and, **d** seed number: box plots showed differences among transplants at below ambient, ambient and above ambient transplant sites. Each box-and-whisker plot represents the observed measures for each population, with the centre bar indicating the median value. Bars with different letters are significantly different (Turkey post hoc tests, *p* < 0.05) and the numbers (n) above each bar of panel represents the sample size

Survival (rhizome sprouting) of transplants at the Ambient (A) the control site and Below Ambient (BA) transplant sites were > 85%, whereas the survival rate decreased to < 50% at Above Ambient (AA) site (Fig. 5a). We observed a significant decrease (Fig. 5b; ANOVA: *F*(2, 109) = 47.77, *p* < 0.001) in the height of *P. sikkimensis* outside of their range limit at BA and AA sites compared to A site. Post hoc comparisons using the Tukey HSDtest [36] indicates that the mean scores for the plant height at three transplant conditions was significantly different (BA: M = 22.41, SD = 10.96; A: M = 29.84, SD = 7.33; AA: M = 9.36, SD = 5.96). Similarly, flower number, representing the initial stage of reproductive fitness, also showed a significant decrease (Fig. 5c; ANOVA: *F* (2, 58) = 40.7, *p* < 0.001) outside the species range limit. Post hoc comparisons using the Tukey HSDtest [36] indicates that the mean scores for the flower number decrease significantly at BA and AA condition compared to A condition (BA: M = 6.08, SD = 2.92; A: M = 17.10, SD = 6.39; AA: M = 6.47, SD = 3.12). However, reproductive fitness represented by average seed production by transplants, was approximately seven seeds per individual at A and BA site, whereas the seed production dropped to four seeds per individual at AA site (Fig. 5d; ANOVA: *F* (2, 26) = 3.39, *p* = 0.05). Post hoc comparisons using the Tukey HSDtest [36] indicates that the mean scores for the seed production decreases significantly at AA (BA: M = 7.25, SD = 2.49; A: M = 7.50, SD = 3.00; AA: M = 4.66, SD = 2.12). Although seed production per individual was higher at A and BA site, the number of individuals producing seeds was less at BA site relative to A site. At A site 12 individuals produced seeds whereas at BA site only 8 individuals produced seeds. Similarly, at AA site, 9 individuals produced seeds. Taken together, we observed an overall decrease in fitness component of *P. sikkimensis* outside their present range limit (Fig. 4a-d), relative to range centre.

## Discussion

Our gene expression analysis demonstrated that plastic gene expression variations have contributed to adaptation in high altitude Himalayan plant species (*Primula sikkimensis*) to different stresses in its natural environment. We identified a large number of genes with plastic expression differences between Ambient versus Below Ambient and Above Ambient conditions. The genes and pathways identified are good candidates for targeted studies of the role of variation in gene expression of a high altitude species to both the hot and cold temperature stress in its natural environment.

### Are mechanisms of stress response conserved?

The below ambient and above ambient transplant sites are located beyond the altitudinal range limit of *P. sikkimensis,* with a temperature differences of approximately 2–3 °C (hotter) and approximately 1–6 °C (colder). Therefore, we compared the significant DEGs of *P. sikkimensis* from the BA vs. A comparison with heat stress genes of *Arabidopsis thaliana* using Gene Expression Omnibus (GEO), at National Center for Biotechnology Information (NCBI). Similarly, the genes from the AA vs. A comparisons were compared to the cold temperature stress genes of *A. thaliana.* Out of 109 significant DEGs of BA vs. A, 83 genes (76%) showed similarity with *A. thaliana* heat stress genes and out of the 85 genes from the AA vs. A comparison 56 genes (65.9%) were similar to *A. thaliana* cold stress genes (Thermal stress (hot): BA vs. A = 76% and (cold): AA vs. A = 65.9%). This supports previous work which suggests that the transcriptomic response to temperature stress might be highly conserved across plant species [37]. The plants at BA site with a higher temperature condition differentially up-regulated more genes than plants at AA site with a cold temperature condition; possibly indicating that expression of an elevated number of genes is necessary for the maintenance of *P. sikkimensis* individuals under heat stress conditions. This suggests that the high-temperature conditions, rather than the cold temperature conditions cause greater differences in the gene expression pattern of *P. sikkimensis* in our study.

### How are below and above ambient different?

Plants are susceptible to adverse environmental conditions. Abiotic stresses such as extreme temperatures, drought, and high UV are some of the typical environmental stressors that can damage physiological functions, and reduce growth and yield of plants [38–40]. In plant communities, environmental stress can be a major source of plant mortality because plants are unable to escape from environmental stress through migration. Constant increases in ambient temperature are considered to be one of the most detrimental environmental stresses affecting plant growth and development [41]. Heat stress is not unique to plants and is also found in other organisms [42]. Heat stress at the molecular level causes an alteration in expression of genes involved in direct protection from high-temperature stress. These include genes responsible for the expression of osmoprotectants, detoxifying enzymes, transporters and regulatory proteins [13]. In our study, *cytochrome P450, Pyridoxal phosphate-dependent decarboxylase, ubiquitin, transcriptional factor B3, HSPs, glycoside hydrolase family 16, NAD-dependent epimerase/dehydratase, haem peroxidize* are some significant DEGs up-regulated in high-temperature conditions at BA transplant site. Similarly the *cytochrome P450, Pyridoxal phosphate, ubiquitin,* and *glycoside hydrolase family* are some of the genes which have been extensively studied in other plants in response to heat stress [43]. On the other hand *Heat shock proteins* (*Hsp20, Hsp70*), *calcium-dependent protein kinase, glutamine aminotransferase* are some significant DEGs down-regulated in high-temperature conditions at BA site (Fig. 1a). These results revealed that most of the genes involved in plant growth and development were up-regulated under BA conditions in *P. sikkimensis* whereas genes involved in signalling and stress-induced proteins (*HSPs*) were down-regulated. *HSPs* are proteins found in plant and animal cells in responsive to heat stress [44, 45]. *HSPs* generally functions as molecular chaperones, and are divided into *HSP20, 40, 60, 70, 90, 100* and *small HSP* (*sHSPs*) [46]. *HSPs* have been shown to increase levels of gene expression when plants are exposed to elevated temperature [47]. However, our result revealed that *HSP20* and *HSP70* were down regulated by heat stress at BA site. As *HSPs* have been shown to be expressed more under heat stress over short time periods [48, 49] it seemed that in our study *HSP20* and *HSP70* genes might had responded for short time period after transplanting plants under heat stress at BA site but decreased with time.

Cold stress also adversely affects plant growth, development, and reproduction. Cold acclimation in plants involves reprogramming of gene expression [50]. Gene expression is induced by cold stress [51, 52] in a number of genes. These genes are thought to be involved in stress tolerance. In case of *Arabidopsis*, the *protein kinases* and *transcription factors* are some of the genes that are up-regulated in response to low temperatures [53]. In our study, *Serine/threonine-protein kinase, phosphoinositide-binding, bifunctional inhibitor/plant lipid transfer protein/seed storage, transcription factor GRAS, DNA-binding WRKY* are up-regulated in cold temperature conditions at AA site (Fig. 1b). These results revealed that most of the genes involved in signalling, defence and membrane transport/permeability were up-regulated under AA conditions in *P. sikkimensis*. It is evident from the pathway analysis that various genes involved in metabolic processes, secondary metabolism, and flavonoid biosynthesis were differentially expressed in BA, whereas genes involved in photosynthesis and plant hormones signalling were differentially expressed in AA site (Fig. 3).

### Fitness variation within and beyond the range limit of *P. sikkimensis*

We observed a decline in rhizome sprouting of transplant individuals at the AA site (< 50% germination rate), whereas at A and BA sites rhizome sprouting was greater than 85%. The decreased in rhizome sprouting of *P. sikkimensis* at AA transplant site may be attributed to cold temperature, as the temperature is an important environmental variable regulating the sprouting of plant rhizomes [54]. Similar to our study, other studies also found the cold temperature to be an important factor in reducing and/or delaying the rhizome sprouting [54, 55]. For example, temperature range of 25–35 °C was optimum for the sprouting of turmeric (*Curcuma longa* L.) rhizomes, and sprouting did not occur below 10 °C [56]. We observed a decrease in plant height and flower number outside species range limit at BA and AA sites. However, the seed production (as a measure of reproductive fitness) was seven seeds per individual at both BA and A site but decreased to four seeds per individual at AA site. The higher reproductive fitness of the BA transplants compared to AA transplants was contrary to what we expect from the cold adaptive *P. sikkimensis* plants. According to the past distributional record of *P. sikkimensis* [57], the species was previously present at much lower elevation than its present lower elevational limits. Therefore, it is possible that the lower elevational limit of *P. sikkimensis* is not determined purely by abiotic factors, but that biotic factors are also be playing a role. These findings suggested that *P. sikkimensis* will favour hot temperature conditions for its germination and reproduction rather than cold conditions, if climate change proceeds in the high altitude of Sikkim Himalaya. However, expression of an elevated number of genes was necessary for the maintenance of *P. sikkimensis* individuals at the hotter temperature conditions as observed in case of BA transplant condition.

## Conclusions

### Plasticity in gene expression

Our study documented many differences in fitness related traits and gene expression associated with thermal stresses which suggest that *P. sikkimensis* undergoes a great deal of plasticity in its transcriptomic profiles*.* Transcriptomic plasticity of this species may facilitate the phenotypic plasticity in morphological and fitness related traits. Comparing the transcriptome profile of *P. sikkimensis* within and beyond the altitudinal range limit of species provided an opportunity to test for the plastic transcriptomic responses of species to stressful environmental condition specifically the thermal stresses. More importantly, transcriptome studies in naturally varying environments show that observed transcriptomic patterns may differ from those seen in controlled experimental conditions. Naturally varying environments may provide a better indication of responses of high elevation plants to ongoing climate change. However, despite the plastic responses of *P. sikkimensis’* transcriptome, the transplant experiment resulted in reduced growth fitness and a decrease in the number of seed producing individuals under temperature stress conditions at BA and AA sites. This decrease indicates the vulnerability of species to future climate change. Future studies combining transcriptomic and genomic data may help in determining the evolutionary significance of transcriptomic variation responses to environmental stress and provide insights into plastic and evolutionary responses to climate change.

## Methods

### Transplant experiment0020

In order to conduct a direct test for adaptive significance of a phenotypic change [58, 59], we transplanted individuals of *Primula sikkimensis* in different environments. Our study was conducted along the elevation gradient of Lachen valley, Sikkim Himalaya (27 °C 62′N, 88 °C 63′E), India in three experimental sites (Additional file 1 Figure S1). The sites were classified as: ambient (A: 3951 (meters above sea level) m a.s.l.), below ambient (BA: 3256 m a.s.l.) and above ambient (AA: 4687 m a.s.l.) after validating elevation range of the species in field (Additional file 6 Table S1). Ambient site was within the altitudinal range limit of focal *P. sikkimensis* species and serves as a control for the experiment. The ambient site is located at an altitude of 3951 m a.s.l. locally knows as Thangu village with an average day temperature of 15 °C and average night temperature of 10 °C during the peak flowering seasons from June to August. Ambient site is also the source population of the *P. sikkimensis* rhizomes selected for the transplant experiment. Below ambient transplant site was approximately 100 m below the lower most elevational range limit of *P. sikkimensis* (3355 m a.s.l.), and the above ambient site was approximately 100 m above the upper elevational range limit of species (4598 m a.s.l.). There is a change in mean annual temperature of 1.5–3 °C at both the below ambient and above ambient transplant sites with respect to its lower most and uppermost elevational range limits, representing stressful environmental conditions (Additional file 2 Figure S2). According to the Intergovernmental Panel on Climate Change (IPCC) report the observed change in temperature from 1901 to 2012 in the Himalayas was 2–3 °C, therefore we designed our experiment to mimic this ongoing environmental change [60, 61]. A total of 300 rhizomes of *P. sikkimensis* were collected from the ambient site for the transplant experiment and were grown individually in a single pot of diameter 22.5 cm and height 30 cm. We kept 100 pots at the ambient site as a control for the experiment, and translocated 200 pots to the below and above ambient sites (100 at each site). We used soil from the ambient site to keep the variation in microfloral properties of the soil constant across transplant sites. The experiment was conducted from March 2013 to September 2014, and the fitness observations of transplants were made starting from March to September of 2014 (rhizome sprouting to reproductive phase). iButtons (hygrochron temperature data logger), were placed at each transplant site to measure the temperature at 2-h intervals from March to September 2014. Our temperature data showed a typical decreasing trend of temperature with increasing elevation as expected [62].

### Transcriptome analysis

#### Plant material, RNA extraction, cDNA library synthesis and Illumina sequencing

Fresh leaf samples of *P. sikkimensis* growing at A, BA, and AA transplant sites were used for transcriptome studies. We collected leaf tissue samples in triplicates (one leaf sample * 5 individuals) randomly from each of the three transplant sites (A, AA, BA) in the same day and the samples were frozen in liquid nitrogen on-site and stored in − 80 °C till RNA isolation.

Total RNA was isolated from leaf samples collected in triplicates from three experimental conditions, using modified RNA isolation protocol [63]. RNA integrity was measured on 1% formaldehyde agarose gel by monitoring distinct 28S and 18S rRNA bands. Purity and concentration of isolated RNA were assessed by monitoring A260/A280 using NanoDrop spectrophotometer 2000C and Bioanalyzer (Additional file 7 Table S2). RNA samples (three RNA samples * three experimental conditions) with RNA Integrity Number (RIN) greater than eight were used for library preparation and sequencing. Transcriptome sequencing was performed using Illumina HiSeq1000 sequencing technology at the Next Generation Genomic Facility at the Centre for Cellular and Molecular Platforms (C-CAMP), Bangalore. The cDNA library for transcriptome sequencing was prepared using TruSeq RNA sample preparation kit V2 from Illumina, as per the manufacturer’s recommendations. The cDNA library was then sequenced using Paired-End 100 base pair chemistry using TruSeq PE cluster kit V3-cBot-HS and TruSeq SBS kit V3-HS for sequencing on the Illumina HiSeq 1000 platform following the manufacturer’s recommended protocols.

#### Read processing and de novo transcriptome assembly

FastQC and FASTX-Toolkit was used for quality checking and pre-processing of raw reads [61]. Read quality was checked and visualized with FastQC [64] and reads with a Phred scaled quality score of less than Q20 was removed. Reads were sorted using FASTX - Toolkit (Hannon Lab) and trimming of low-quality reads was performed using FASTX – Trimmer.

De novo transcriptome assembly of *P. sikkimensis* from all three conditions was performed independently by combining the filtered reads of biological triplicates. The reference transcriptome assembly was generated by combining reads of biological triplicates from all three conditions i.e., A, BA and AA. *Denovo* assembly was performed for all K-mers from 19 to 61 at an interval of two using Trinity [65]. Contigs shorter than 100 base pair (bp) were eliminated.

#### Transcript differential abundance calculation

Transcript abundance quantification was performed in terms of fragments per kilobase of exon per million fragments mapped (FPKM). A reference transcriptome (combining all three conditions) of non-redundant combined assembled transcriptome sequences at 90% sequence similarity by CD-HIT-EST [66] was taken. The reads from all the samples were mapped back to the reference transcriptome by using TopHat2 [67] at default parameters. Technical duplicates were merged using SAMTOOLS. Cufflinks was used to generate a GTF file for each gene model from the combined transcriptomic sequences [68]. The FPKM values for each transcript in all the samples were determined. The differential abundance of transcripts among different samples/sites was calculated by the cuffdiff 2 tool [69]. The complete workflow is provided in Additional file 3 Figure S3. Gene Ontology (GO) is an international standardized gene functional classification system which describes properties of genes and their products in any organism. GO is standardized gene functional classification system and it has three ontologies: cellular component, molecular function, and biological process. Functional annotation in terms of GO [70] and gene family of the transcripts was done by using TRAPID [71], an online server. TRAPID gives the option to search against the available databases viz., PLAZA 2.5 and OrthoMCLDB version 5. After getting GO annotation, the functional enrichment of the transcripts was also performed and abundant transcripts in each condition were plotted by using WEGO tool [72](Additional file 4 Figure S4).

#### Identification of differentially expressed genes (DEGs) and functional annotation

Analyses of DEGs includes the screening of genes that were differentially expressed among two pair-wise comparisons (BA vs. A and AA vs. A), and GO functional enrichment and KEGG (Kyoto Encyclopaedia of Genes and Genomes) pathway enrichment analysis for these DEGs. We used a value of log_2_ (fold change) ≥ 2 and mean expression value of log_10_ (*p*-value) < 0.05, as the threshold to judge the significance of gene expression difference [34]. We used Blast2GO [73] to get GO annotation for significant DEGs of two pair-wise comparisons. After getting GO annotation for every DEGs, we used WEGO [72] to do GO functional classification. KEGG is a pathway-related database and pathway enrichment analysis identifies significantly enriched pathways in DEGs [74], and KOBAS [75] was used to test the statistical significance of the enrichment of DEGs in KEGG pathways [35].

#### Real-time PCR analysis

Total RNA was isolated from A, BA, and AA samples as described above for transcriptome analysis. RNA extracts were treated with DNase I, amplification grade (Invitrogen, USA) to remove DNA contamination. Complementary DNAs (cDNAs) were synthesized using SuperScript III cDNA synthesis kit (Invitrogen, USA) as per the protocol.

The differentially expressed genes were selected randomly for qRT-PCR from A, BA, AA conditions based on their FPKM values (Additional file 5 Figure S5). The primers for all the genes were designed using the Primer3Plus software [76] as listed in Table 2. All the PCR reactions were performed in triplicatesin 10 μl reaction mixture containing diluted cDNA samples as template, 2× SYBR® Green Master Mix (Applied Biosystems, USA), and 200 nM each of forward and reverse gene-specific primers (Table 2). The reactions were performed in StepOnePlus™ Real-Time PCR System (Applied Biosystems, USA) using the following program: initial denaturation at 94 °C for 10 min, followed by 40 cycles of amplification (94 °C for 30s, 60 °C for 30s, and 72 °C for 30 s) and final melt curve analysis was performed. Transcript levels of all the genes were normalized with an internal control reference *26S rRNA* gene [77]. The relative expression ratio of each gene was calculated using the comparative Ct value method as described previously [78]. Here, the transcript levels represented are relative quantitation (RQ) values of gene expression. Expression is shown after normalization to *26S rRNA* gene. Values were calculated using the ΔΔCT method, and the error bars represented as RQ_MIN_ and RQ_MAX._

### Survival, growth and reproductive fitness of transplant individuals among three transplant sites

We recorded the number of rhizomes sprouted from the transplanted rhizomes at each site. Height (cm) of transplants was measured as a representative of growth fitness. Flower number and seed number per transplanted individual in each transplant site were quantified as a measure of reproductive fitness. The differences in growth and reproductive fitness of transplant individuals between the three transplant sites (A, BA and AA) were assessed using ANOVA. Here we consider all the transplant individuals that manage to reach growth and/or reproductive maturity including the individuals which we used for RNA-seq analysis. When significant differences were observed, the ANOVA was followed by the Tukey posthoc tests [36] for pair-wise comparisons after Bonferroni correction.

## Supplementary information

**Additional file 1: Figure S1.** Transplant sites.

**Additional file 2: Figure S2.** Temperature across transplant sites.

**Additional file 3: Figure S3.** Workflow of differential expression of genes.

**Additional file 4: Figure S4.** WEGO.

**Additional file 5: Figure S5.** Heatmap plot for 25 random DEGs.

**Additional file 6: Table S1.** Details of three transplant sites.

**Additional file 7: Table S2.** Yield and quality of RNA samples.

**Additional file 8: Table S3.** Details list of significant DEGs in Below ambient versus Ambient and Above ambient versus Ambient transplant condition.

**Additional file 9: Table S4.** Pathway details.

## Data Availability

The data supporting the results of this article are available in the National Centre for Biotechnology Information (NCBI) [Accession number: SRP150603 (SRX4219916, SRX4219915, SRX4219914, SRX4219913, SRX4219912, SRX4219911, SRX4219910, SRX4219909, SRX4219908)]. All the supporting data are available in the electronic supplementary material.

## Abbreviations

A: Ambient
AA: Above ambient
BA: Below ambient
DEG: Differentially expressed gene
GO: Gene Ontology
KEGG: The Kyoto Encyclopedia of Genes and Genomes
m a.s.l.: Meters above sea level
bp: Base pair
GEO: Gene Expression Omnibus
NCBI: National Centre for Biotechnology Information
FPKM: Fragments per kilobase of exon per million fragments mapped
RIN: RNA Integrity Number
IPCC: Intergovernmental Panel on Climate Change
RQ: Realtive quantification
