# Supplementary figures and images for: Transcriptome analysis reveals plasticity in gene regulation due to environmental cues in Primula sikkimensis, a high altitude plant species
Source: BMC Genomics. 2019 Dec 17;20:989. doi: 10.1186/s12864-019-6354-1 (PMC6916092; doi:10.1186/s12864-019-6354-1)

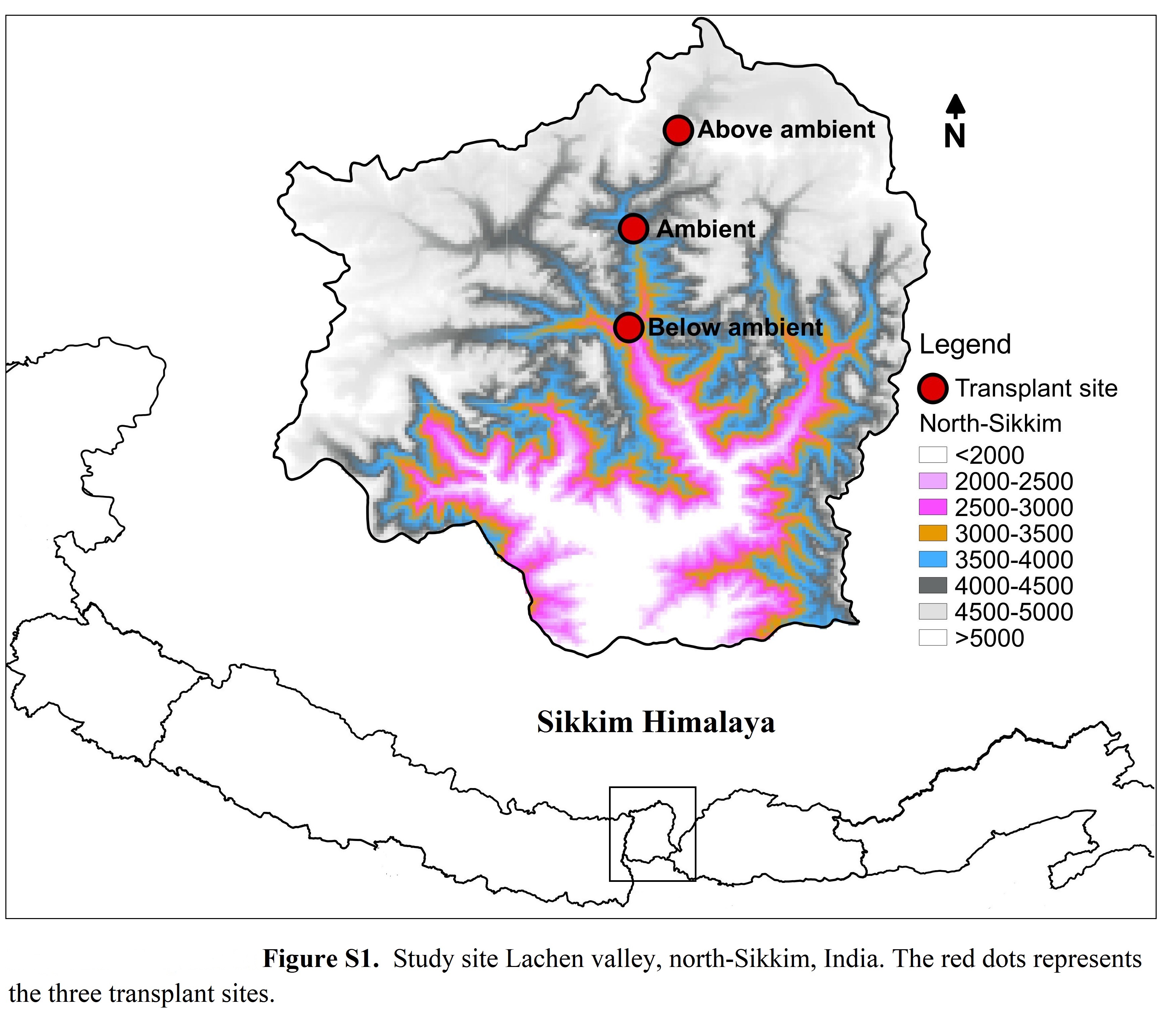

Supplement: Supplementary file 1 — Additional file 1: Figure S1. Transplant sites. [file 12864_2019_6354_MOESM1_ESM.jpg]

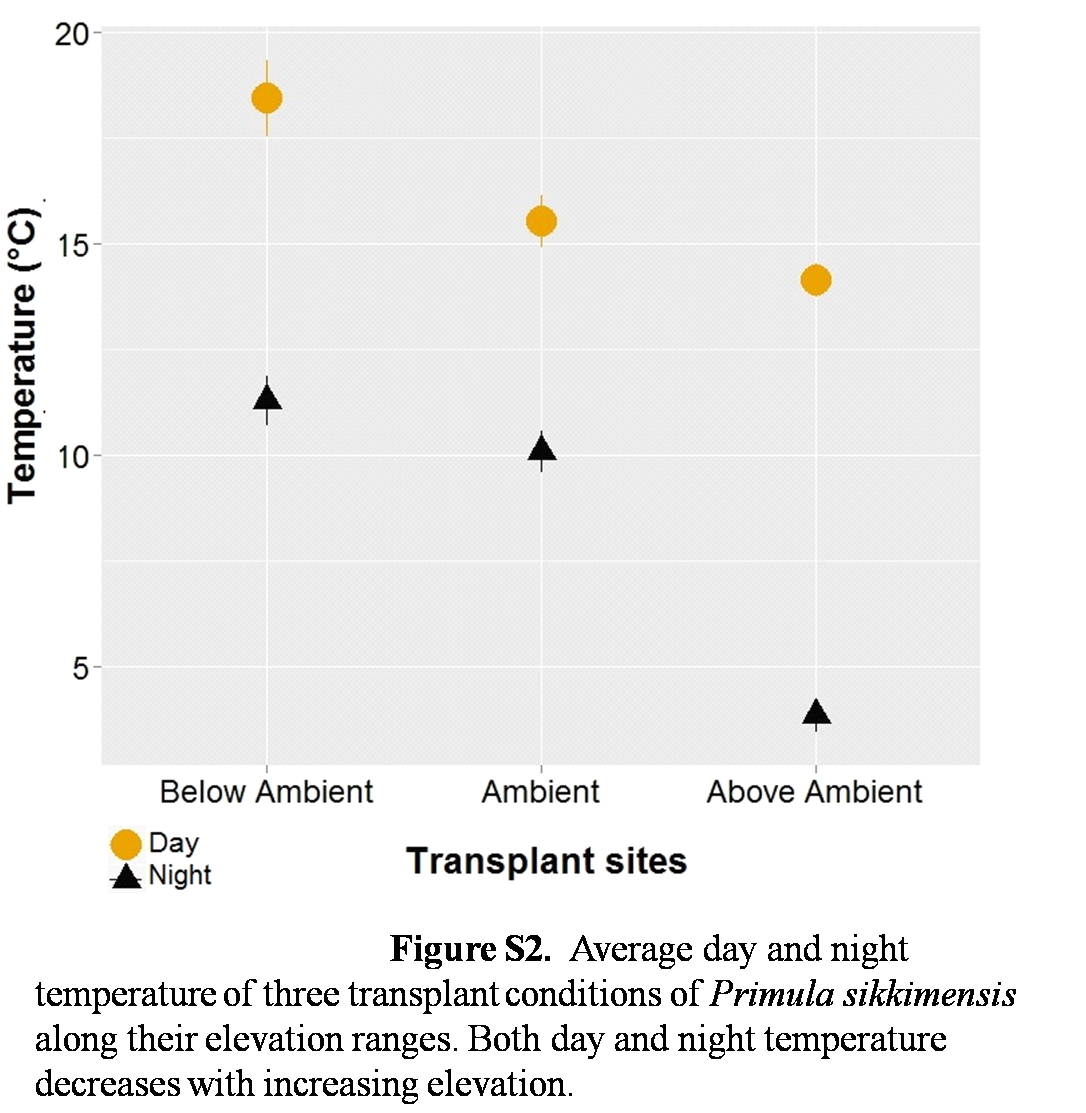

Supplement: Supplementary file 2 — Additional file 2: Figure S2. Temperature across transplant sites. [file 12864_2019_6354_MOESM2_ESM.png]

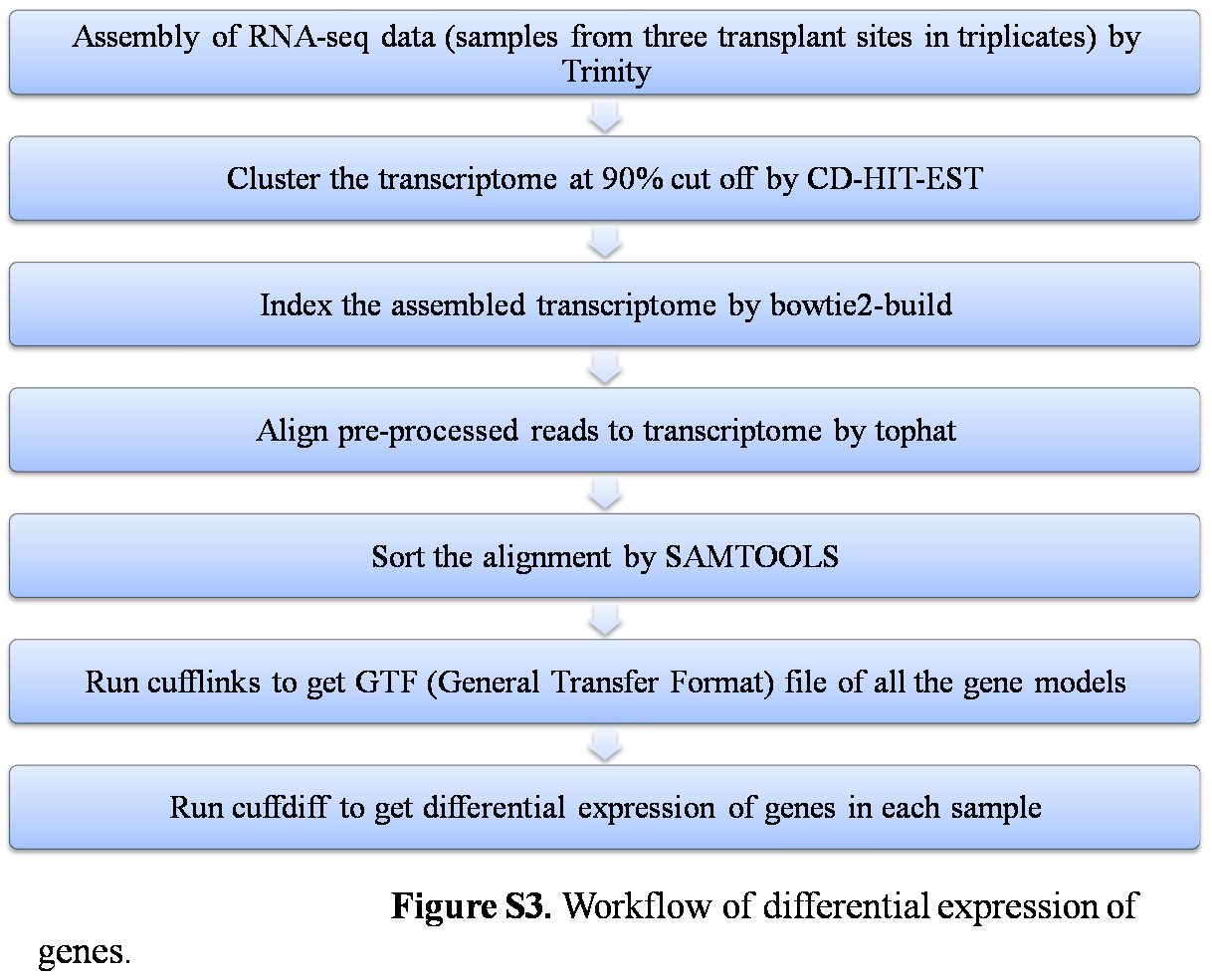

Supplement: Supplementary file 3 — Additional file 3: Figure S3. Workflow of differential expression of genes. [file 12864_2019_6354_MOESM3_ESM.png]

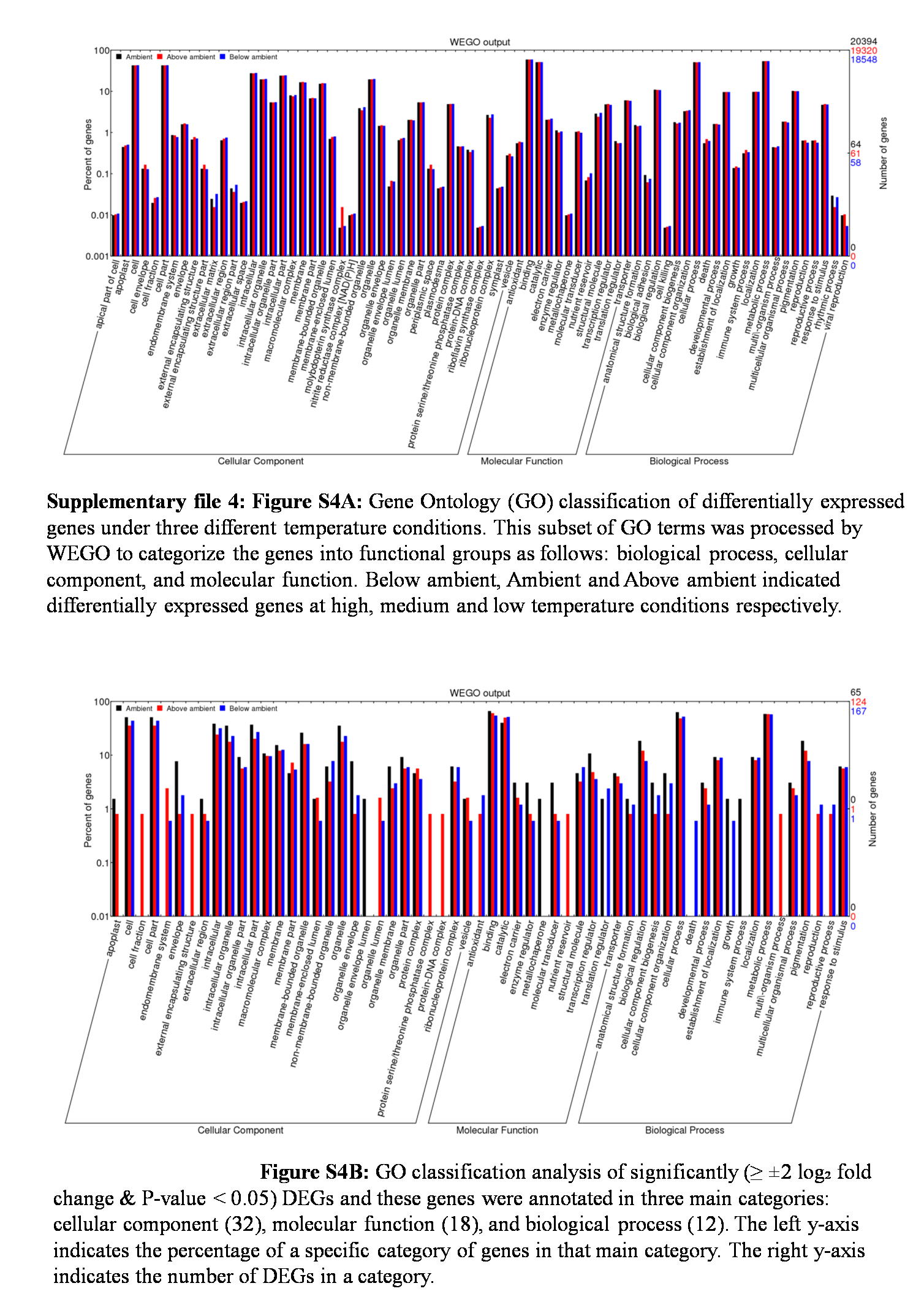

Supplement: Supplementary file 4 — Additional file 4: Figure S4. WEGO. [file 12864_2019_6354_MOESM4_ESM.png]

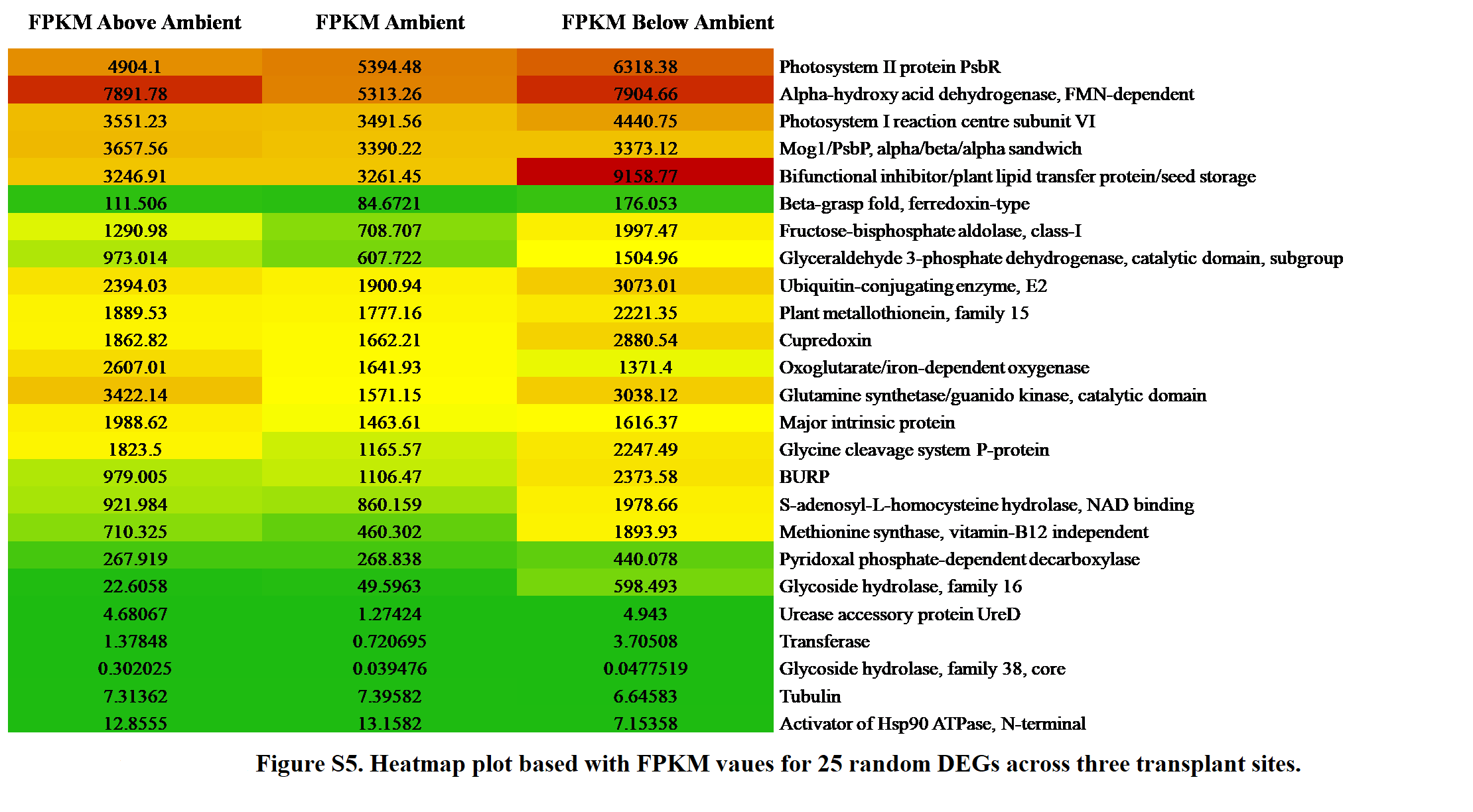

Supplement: Supplementary file 5 — Additional file 5: Figure S5. Heatmap plot for 25 random DEGs. [file 12864_2019_6354_MOESM5_ESM.png]
