# Supplementary material for: Transcriptome analysis reveals plasticity in gene regulation due to environmental cues in Primula sikkimensis, a high altitude plant species
Source: BMC Genomics. 2019 Dec 17;20:989. doi: 10.1186/s12864-019-6354-1 (PMC6916092; doi:10.1186/s12864-019-6354-1)
Supplement: Supplementary file 6 — Additional file 6: Table S1. Details of three transplant sites. [file 12864_2019_6354_MOESM6_ESM.docx]

**Table S1.** Details of three transplant sites Ambient (A), Below ambient (BA), and Above ambient (AA), along with the average temperature values of the peak growing season (June to August 2014).

| **Transplant site** | **Below ambient (BA)** | **Ambient (A)** | **Above ambient (AA)** |
| --- | --- | --- | --- |
| Site name | Zema III | Thangu | Gayagaon |
| Altitude (m a.s.l.) | 3256 | 3951 | 4687 |
| Latitude | N2746.936 | N2753.877 | N280.071 |
| Longitude | E0882.781 | E08832.276 | E08835.616 |
| Temp. max ͫ day (°C) | 35.625 | 27.5 | 24.5 |
| Temp max ͫ night (°C) | 13.624 | 16 | 6.5 |
| Temp. min ͫ day (°C) | 8.103 | 9 | 3.5 |
| Temp min ͫ night (°C) | 5.088 | 4 | -3.5 |
| Temp. avg. day (°C) ±SE | 18.45119±0.23 | 15.56483±0.13 | 14.13852±0.02 |
| Temp. avg. night (°C)±SE | 11.29786±0.06 | 10.09588±0.07 | 3.877395±0.06 |
