# Supplementary material for: Transcriptome analysis reveals plasticity in gene regulation due to environmental cues in Primula sikkimensis, a high altitude plant species
Source: BMC Genomics. 2019 Dec 17;20:989. doi: 10.1186/s12864-019-6354-1 (PMC6916092; doi:10.1186/s12864-019-6354-1)
Supplement: Supplementary file 7 — Additional file 7: Table S2. Yield and quality of RNA samples. [file 12864_2019_6354_MOESM7_ESM.docx]

**Table S2.** Yield and quality of RNA samples

| S. No | Plant part | Sample Name | Sample Concentration  (ng/ul) | Volume (ul) | Total Concentration (ug) | RNA Integrity (RIN) Value |
| --- | --- | --- | --- | --- | --- | --- |
| 1 | Leaf | Ambient 1 | 380 | 15 | 5.7 | 5.8 |
| 2 | Leaf | Ambient 2 | 678 | 15 | 10.1 | 5.9 |
| 3 | Leaf | Ambient 3 | 994 | 10 | 9.9 | 4.3 |
| 4 | Leaf | Above ambient 1 | 432 | 18 | 7.7 | 5.9 |
| 5 | Leaf | Above ambient 2 | 346 | 20 | 6.9 | 5 |
| 6 | Leaf | Above ambient 3 | 992 | 22 | 21.8 | 4.8 |
| 7 | Leaf | Below ambient 1 | 121 | 10 | 1.2 | 3.6 |
| 8 | Leaf | Below ambient 2 | 252 | 10 | 2.5 | 2.6 |
| 9 | Leaf | Below ambient 3 | 698 | 19 | 13.2 | 4.4 |
