# Supplementary material for: Transcriptome analysis reveals plasticity in gene regulation due to environmental cues in Primula sikkimensis, a high altitude plant species
Source: BMC Genomics. 2019 Dec 17;20:989. doi: 10.1186/s12864-019-6354-1 (PMC6916092; doi:10.1186/s12864-019-6354-1)
Supplement: Supplementary file 8 — Additional file 8: Table S3. Details list of significant DEGs in Below ambient versus Ambient and Above ambient versus Ambient transplant condition. [file 12864_2019_6354_MOESM8_ESM.docx]

**Table S3.** List of significant differentially expressed genes in two pair-wise comparisons of transplant conditions. A: Below ambient versus Ambient; B: Above ambient versus Ambient condition. Genes unique to each condition are highlighted in pink color.

| **A: Below ambient versus Ambient: Significant DEGs up-regulated** | | | | | |
| --- | --- | --- | --- | --- | --- |
| **CUFF_ID** | **GENE NAME** | **GO Functional Annotation** | **Below Ambient** | **Ambient** | **Fold Change** |
| c19109_g1_i1 | ABC transporter-like | purine ribonucleotide binding, ribonucleotide binding | 10.8654 | 2.70791 | 2.00449 |
| c16979_g1_i1 | Inorganic pyrophosphatase | Inorganic pyrophosphatase | 413.546 | 102.109 | 2.01795 |
| c57533_g1_i1 | Nucleotide sugar dehydrogenase | Nucleotide sugar dehydrogenase | 114.063 | 28.123 | 2.02001 |
| c18941_g1_i2 | Tubulin | Thaumatin, pathogenesis-related | 49.9622 | 12.1902 | 2.03512 |
| c22865_g1_i1 | Methionine synthase, vitamin-B12 independent | Methionine synthase, vitamin-B12 independent | 1893.93 | 460.302 | 2.04073 |
| c19391_g3_i1 | Xanthine/uracil/vitamin C permease | transmembrane transporter activity, transmembrane transport | 122.216 | 29.2177 | 2.06451 |
| c23887_g1_i1 | AMP-dependent synthetase/ligase | metabolic process, ligase activity | 320.571 | 76.4747 | 2.06759 |
| c26627_g2_i1 | Nonaspanin (TM9SF) | intracellular organelle, intrinsic to membrane | 3.94528 | 0.931864 | 2.08194 |
| c7205_g1_i1 | Tudor subgroup | organic acid biosynthetic process, multicellular organismal process, macromolecule localization | 0.335931 | 0.0793461 | 2.08194 |
| c1434_g1_i1 | Curculin-like (mannose-binding) lectin | external encapsulating structure, plant-type cell wall | 147.624 | 34.5393 | 2.09561 |
| c22358_g1_i1 | DAHP synthetase, class II | 3-deoxy-7-phosphoheptulonate synthase activity, small molecule biosynthetic process | 820.461 | 186.419 | 2.13789 |
| c1680_g1_i1 | TRAM/LAG1/CLN8 homology domain | intrinsic to membrane | 7.59424 | 1.719 | 2.14334 |
| c18965_g1_i1 | RNA-binding S4 | cellular macromolecule metabolic process, small ribosomal subunit | 2.19565 | 0.495034 | 2.14905 |
| c21917_g1_i3 | Six-bladed beta-propeller, TolB-like | intracellular membrane-bounded organelle, purine ribonucleotide binding | 14.1912 | 3.16609 | 2.16422 |
| c20037_g3_i1 | Actin/actin-like | adenyl ribonucleotide binding, purine nucleotide binding | 174.93 | 38.7709 | 2.17373 |
| c16420_g1_i1 | BURP |  | 117.634 | 26.0141 | 2.17694 |
| c14384_g1_i1 | dTDP-4-dehydrorhamnose reductase | coenzyme binding, cellular polysaccharide metabolic process, cellular carbohydrate metabolic process, extracellular polysaccharide metabolic process | 648.427 | 143.162 | 2.1793 |
| c19444_g1_i1 | Thiolase-like, subgroup | small molecule biosynthetic process, oxoacid metabolic process, monocarboxylic acid metabolic process | 36.2105 | 7.99172 | 2.17983 |
| c5125_g1_i1 | Cytochrome b561/ferric reductase transmembrane | Cytochrome b561/ferric reductase transmembrane | 38.1055 | 8.36553 | 2.18747 |
| c14622_g1_i1 | L-Aspartase-like | nitrogen compound metabolic process, cellular aromatic compound metabolic process | 78.8383 | 17.1616 | 2.19971 |
| c15383_g1_i1 | Peptidase T2, asparaginase 2 | hydrolase activity, acting on carbon-nitrogen (but not peptide) bonds, in linear amides | 408.244 | 88.6085 | 2.20392 |
| c25267_g1_i1 | Phospholipid/glycerol acyltransferase | Phospholipid/glycerol acyltransferase | 299.177 | 64.407 | 2.21571 |
| c15207_g1_i1 | Tetratricopeptide repeat | binding | 5.44733 | 1.17137 | 2.21736 |
| c20800_g2_i1 | S-adenosylmethionine synthetase | S-adenosylmethionine synthetase | 1066.91 | 223.369 | 2.25594 |
| c37545_g1_i1 | Thymidylate synthase | nitrogen compound metabolic process, pyrimidine nucleotide biosynthetic process | 13.412 | 2.77188 | 2.27458 |
| c16149_g1_i1 | Phospholipase/carboxylesterase | hydrolase activity, acting on ester bonds | 64.7827 | 13.3083 | 2.28328 |
| c6362_g1_i1 | S1/P1 nuclease | cellular macromolecule catabolic process, nitrogen compound metabolic process | 3.95497 | 0.801962 | 2.30206 |
| c12016_g1_i1 | Transcriptional factor B3 | egulation of gene expression, nitrogen compound metabolic process | 7.57929 | 1.52656 | 2.31178 |
| c23558_g1_i1 | PAR1 |  | 84.1035 | 16.8569 | 2.31883 |
| c47207_g1_i1 | Auxin efflux carrier | establishment of localization, integral to membrane | 2.05715 | 0.40815 | 2.33347 |
| c19483_g1_i1 | Cupin, RmlC-type | nutrient reservoir activity, transition metal ion binding, apoplast | 5163.25 | 1019.85 | 2.33992 |
| c14428_g1_i1 | Carbohydrate/purine kinase | Carbohydrate/purine kinase | 44.2727 | 8.73779 | 2.34108 |
| c9247_g1_i1 | Ammonium transporter | Ammonium transporter | 263.307 | 51.6061 | 2.35113 |
| c54569_g1_i1 | Nucleic acid-binding, OB-fold-like | cellular macromolecule metabolic process, organellar large ribosomal subunit | 1.25322 | 0.24377 | 2.36204 |
| c26101_g1_i1 | GCN5-related N-acetyltransferase | transferase activity, transferring acyl groups other than amino-acyl groups | 1.72191 | 0.334939 | 2.36204 |
| c14795_g1_i1 | Metallophosphoesterase | hydrolase activity | 4.26319 | 0.813308 | 2.39006 |
| c12756_g1_i1 | SCP-like extracellular | extracellular region | 1.30303 | 0.248585 | 2.39006 |
| c18101_g1_i1 | Mob1/phocein | protein binding, binding | 73.6652 | 14.0136 | 2.39416 |
| c15550_g1_i1 | NAD-dependent epimerase/dehydratase | NAD-dependent epimerase/dehydratase | 564.758 | 106.551 | 2.40608 |
| c26519_g2_i1 | Chorismate mutase, type II | Chorismate mutase, type II | 61.3872 | 11.558 | 2.40905 |
| c25896_g2_i1 | Delayed-early response protein/equilibrative nucleoside transporter | Delayed-early response protein/equilibrative nucleoside transporter | 16.3972 | 3.08627 | 2.40951 |
| c26841_g1_i1 | Cystinosin/ERS1p repeat | proteasome complex | 2.47549 | 0.465105 | 2.41208 |
| c3140_g1_i1 | NAD(P)-binding domain | catalytic activity, metabolic process | 299.077 | 53.7615 | 2.47587 |
| c18956_g1_i2 | 3-oxo-5-alpha-steroid 4-dehydrogenase, C-terminal | oxidoreductase activity, acting on the CH-CH group of donors | 28.4934 | 4.88843 | 2.54318 |
| c25326_g2_i1 | KOW | regulation of gene expression, nitrogen compound metabolic process | 2.11588 | 0.349835 | 2.59651 |
| c15461_g1_i1 | Core-2/I-Branching enzyme | UDP-glycosyltransferase activity, transferase activity | 7.39957 | 1.17921 | 2.64962 |
| c25059_g1_i4 | Succinate dehydrogenase/Fumarate reductase, transmembrane subunit | Succinate dehydrogenase activity, Fumarate reductase, transmembrane subunit | 1.51504 | 0.231939 | 2.70754 |
| c12876_g1_i1 | Chalcone isomerase, subgroup | cellular aromatic compound metabolic process, intramolecular lyase activity, phenylpropanoid biosynthetic process | 874.859 | 131.829 | 2.73038 |
| c61887_g1_i1 | Toll-like receptor, leucine rich repeat-containing | Toll-like receptor molecule, leucine rich repeat-containing | 0.945611 | 0.142132 | 2.73401 |
| c25059_g4_i2 | Protein precursor Ycf15, putative, chloroplast | Protein precursor Ycf15, putative hypothetical, chloroplast | 1.13774 | 0.167956 | 2.76001 |
| c25365_g1_i1 | Multi antimicrobial extrusion protein MatE | Multi antimicrobial extrusion protein - MatE | 17.8064 | 2.43149 | 2.87248 |
| c11128_g1_i1 | Pollen Ole e 1 allergen/extensin | Pollen Ole e 1 allergen and extensin | 1191.74 | 159.804 | 2.8987 |
| c3551_g1_i1 | Cytochrome P450 | vitamin B6 binding, biosynthetic process, transferase activity, transferring nitrogenous groups | 29.8519 | 3.5652 | 3.06577 |
| c8623_g1_i1 | Nucleoporin protein Ndc1-Nup |  | 2.18026 | 0.257486 | 3.08194 |
| c21862_g1_i1 | Berberine/berberine-like | Berberine/berberine-like protein family | 18.255 | 1.30928 | 3.80144 |
| c15694_g1_i1 | Lipase, GDSL | lipid metabolic process | 57.7939 | 14.4108 | 2.00377 |
| c62082_g1_i1 | FAS1 domain | response to organic cyclic substance | 187.005 | 46.4527 | 2.00924 |
| c10460_g1_i1 | UDP-glucuronosyl/UDP-glucosyltransferase | UDP-glucuronosyl/UDP-glucosyltransferase, transferase activity | 130.865 | 32.3812 | 2.01485 |
| c24476_g1_i1 | Thaumatin, pathogenesis-related | Thaumatin, pathogenesis-related | 275.639 | 67.7731 | 2.02399 |
| c25002_g1_i1 | Cupredoxin | oxidoreductase activity, catalytic activity, transition metal ion binding | 22.6515 | 5.55556 | 2.0276 |
| c12558_g1_i1 | Heat shock protein DnaJ, N-terminal | Heat shock protein DnaJ, N-terminal | 42.328 | 10.238 | 2.04768 |
| c820_g1_i1 | Phosphate-induced protein 1 | Phosphate-induced protein 1 | 44.3316 | 10.7154 | 2.04865 |
| c23313_g1_i1 | Pectinesterase inhibitor | enzyme inhibitor activity, pectinesterase activity, hydrolase activity | 85.8846 | 20.5842 | 2.06086 |
| c22232_g1_i1 | Heavy metal transport/detoxification protein | Heavy metal transport/detoxification protein | 21.2866 | 5.02399 | 2.08304 |
| c24851_g1_i1 | Ribonuclease T2 | endonuclease activity, active with either ribo- or deoxyribonucleic acids and producing 3'-phosphomonoesters, | 669.333 | 151.148 | 2.14676 |
| c26497_g1_i1 | Immunoglobulin E-set |  | 10.4009 | 2.29942 | 2.17736 |
| c6781_g1_i1 | Oligopeptide transporter OPT superfamily | Oligopeptide transporter OPT superfamily | 1.55261 | 0.342274 | 2.18147 |
| c20093_g1_i1 | Haem peroxidase, plant/fungal/bacterial | iron ion binding, cation binding, transition metal ion binding | 11.4155 | 2.32507 | 2.29564 |
| c16281_g1_i1 | Chalcone/stilbene synthase, C-terminal | transferase activity, transferring acyl groups | 1402.77 | 261.412 | 2.42389 |
| c22476_g1_i1 | Late embryogenesis abundant protein, type 2 |  | 6.50822 | 1.10199 | 2.56216 |
| c15827_g1_i2 | Ubiquitin supergroup | Ubiquitin supergroup | 7.1952 | 1.19985 | 2.58418 |
| c316_g1_i1 | Alcohol dehydrogenase superfamily, zinc-containing | Alcohol dehydrogenase superfamily, zinc-containing | 1.22101 | 0.20188 | 2.59651 |
| c2210_g1_i1 | C2 membrane targeting protein |  | 3.30209 | 0.505519 | 2.70754 |
| c26125_g1_i1 | 2-phosphoglycolate phosphatase, eukaryotic | 2-phosphoglycolate phosphatase, eukaryotic in nature | 2.37556 | 0.352487 | 2.75263 |
| c4819_g1_i2 | Oxoglutarate/iron-dependent oxygenase | oxidoreductase enzymatic activity, catalytic activity | 2.84922 | 0.247939 | 3.52251 |
| c55573_g1_i1 | Transcription factor GRAS |  | 3.53777 | 0.297421 | 3.57226 |
| c16448_g1_i1 | Glycoside hydrolase, family 16 | Glycoside hydrolase and family 16 | 598.493 | 49.5963 | 3.59303 |
| c55251_g1_i1 | Polyketide cyclase/dehydrase | Polyketide cyclase/dehydrase enzymatic activity | 7.46936 | 0.503385 | 3.89125 |
| c9426_g1_i1 | Ribosomal protein S21e | cellular macromolecule metabolic process, cellular macromolecule biosynthetic process | 104.413 | 6.71888 | 3.95794 |
| c25489_g1_i1 | Pyridoxal phosphate-dependent decarboxylase | small molecule metabolic process, oxoacid metabolic process | 15.6727 | 0.638367 | 4.61773 |
| c6097_g1_i1 | Peptidoglycan-binding lysin domain | cell wall macromolecule metabolic process | 14.2839 | 0.0606335 | 7.88006 |
|  | | | | | |
| **Below ambient versus Ambient: Significant DEGs down-regulated** | | | | | |
| c20707_g1_i1 | Protein phosphatase 2C-related | Protein phosphatase 2C-related | 0.125972 | 5.51516 | -5.45223 |
| c54193_g1_i1 | Polynucleotidyl transferase, ribonuclease H fold | Polynucleotidyl transferase, ribonuclease H fold | 0.133209 | 5.17575 | -5.28001 |
| c12091_g1_i1 | Zinc finger, SIAH-type | Zinc finger and SIAH-type | 0.0962813 | 3.1042 | -5.01082 |
| c20496_g1_i1 | Glutamine amidotransferase, type II | aspartate family amino acid biosynthetic process | 0.223001 | 4.60882 | -4.36928 |
| c11770_g1_i1 | Serine/threonine-protein kinase domain | Serine/threonine-protein kinase domain family | 0.126638 | 1.57035 | -3.63231 |
| c4296_g1_i1 | Carbonic anhydrase, CAH1-like | Carbonic anhydrase, CAH1-like activity | 5.65331 | 67.5472 | -3.57873 |
| c22372_g1_i3 | Actin-binding, cofilin/tropomyosin type | cytoskeletal protein binding, Actin-binding, cofilin/tropomyosin type | 0.139446 | 1.34492 | -3.26974 |
| c16945_g1_i1 | Pentatricopeptide repeat | leaf vasculature patterning | 0.30989 | 1.92137 | -2.63231 |
| c17987_g1_i2 | Mitochondrial substrate carrier | mitochondrial inner membrane, mitochondrial envelope, establishment of localization | 1.3503 | 7.81394 | -2.53277 |
| c22748_g1_i1 | Thioredoxin-like fold | cellular homeostasis, cellular process, biological regulation | 3.27862 | 16.0578 | -2.29212 |
| c24617_g1_i1 | Terpenoid synthase | magnesium ion binding, cation binding | 3.12679 | 13.3701 | -2.09626 |
| c21833_g1_i1 | Metal-dependent phosphohydrolase, HD domain | small molecule metabolic process, Metal-dependent phosphohydrolase, HD domain | 4.89056 | 20.8887 | -2.09465 |
| c26128_g2_i3 | Malate transporter, aliminium toerance | response to inorganic substance, response to metal ion | 0.436568 | 1.85265 | -2.08531 |
| c25222_g1_i3 | Helicase/SANT-associated, DNA binding | binding, nucleic acid binding | 0.250971 | 1.02969 | -2.03662 |
| c24656_g1_i2 | RNA ligase/cyclic nucleotide phosphodiesterase | RNA ligase/cyclic nucleotide phosphodiesterase | 0.0015953 | 0.864874 | -9.08252 |
| c17395_g1_i1 | Heat shock protein Hsp20 | Heat shock protein Hsp20 etc | 1.2645 | 37.284 | -4.88192 |
| c968_g1_i1 | Barwin-related endoglucanase | nitrogen compound metabolic process, chitin metabolic process | 5.27659 | 90.5819 | -4.10154 |
| c20499_g1_i1 | Calcium/calmodulin-dependent protein kinase-like | Calcium - calmodulin-dependent protein kinase-like | 0.153621 | 2.3177 | -3.91524 |
| c761_g1_i1 | Ribosomal protein S30Ae/sigma 54 modulation protein | intracellular membrane-bounded organelle, cytoplasmic part | 8.84346 | 129.243 | -3.86933 |
| c22270_g1_i1 | Cytochrome P450, E-class, group I | transition metal ion binding, Cytochrome P450, E-class, group I | 0.415938 | 4.57752 | -3.46013 |
| c17775_g1_i2 | Protein kinase, catalytic domain | Protein kinase enyme, catalytic domain | 0.22158 | 1.86231 | -3.07119 |
| c15561_g1_i1 | DNA-binding WRKY | nitrogen compound metabolic process, transcription factor activity | 0.741572 | 4.75953 | -2.68216 |
| c20050_g1_i1 | Signal transduction response regulator, receiver domain | Signal transduction response regulator, receiver domain | 0.828653 | 4.71115 | -2.50724 |
| c23052_g1_i2 | Amino acid transporter, transmembrane | Amino acid transporter and transmembrane protein regulation | 0.0790201 | 0.391951 | -2.31038 |
| c23102_g1_i1 | Glutathione S-transferase/chloride channel, C-terminal | toxin catabolic process, cellular process, secondary metabolic process | 10.5766 | 50.878 | -2.26617 |
| c26655_g1_i3 | Heat shock protein 70 | adenyl nucleotide binding, nucleoside binding | 5.23112 | 25.1608 | -2.26599 |
| c26074_g1_i1 | Glycosyltransferase AER61, uncharacterised | nucleotide binding, transferase activity, transferring glycosyl groups | 1.06582 | 4.46492 | -2.06667 |
| c13691_g1_i1 | Pectate lyase/Amb allergen | carbon-oxygen lyase activity, acting on polysaccharides, carbon-oxygen lyase activity, carbon-oxygen lyase activity, acting on polysaccharides | 6.66866 | 32.6237 | -2.29045 |
|  | | | | | |
| **B: Above ambient versus Ambient: Significant DEGs up-regulated** | | | | | |
| **CUFF_ID** | **GENE NAME** | **GO Functional Annotation** | **Above Ambient** | **Ambient** | **Fold Change** |
| c10028_g1_i1 | Helix-loop-helix DNA-binding domain | regulation of gene expression, gene expression, nitrogen compound metabolic process | 4.8525 | 0.104464 | 5.53766 |
| c2897_g1_i1 | Bifunctional inhibitor/plant lipid transfer protein/seed storage | response to biotic stimulus, macromolecule localization, multi-organism process | 15.8394 | 0.587514 | 4.75276 |
| c25421_g2_i1 | Serine/threonine-protein kinase-like domain | purine ribonucleotide binding, cellular macromolecule metabolic process | 1.36426 | 0.0683946 | 4.31809 |
| c8801_g1_i1 | Zinc finger, C2H2-type | intracellular, zinc ion binding | 4.44976 | 0.244274 | 4.18716 |
| c3730_g1_i1 | C2 calcium-dependent membrane targeting | protein binding, response to heat, response to chemical stimulus | 18.9508 | 2.12571 | 3.15624 |
| c547_g1_i1 | Glycoside hydrolase, family 38, core | cellular carbohydrate metabolic process, | 0.302025 | 0.039476 | 2.93562 |
| c24053_g1_i1 | Clathrin adaptor, phosphoinositide-binding, GAT-like | protein binding, coated membrane, cellular component biogenesis | 9.09273 | 1.22823 | 2.88813 |
| c26581_g1_i1 | Extracellular solute-binding protein, family 3 | transporter activity, extracellular ligand-gated ion channel activity | 4.01943 | 0.562883 | 2.83608 |
| c2324_g1_i1 | RNA-directed DNA polymerase (reverse transcriptase), related | organelle envelope, plastid envelope, response to cold | 2.31813 | 0.334883 | 2.79123 |
| c20889_g1_i1 | Ribosomal protein S11 | gene expression, cellular macromolecule metabolic process, translation | 4.41884 | 0.672163 | 2.71678 |
| c4771_g1_i1 | Cyclin-like F-box |  | 1.36858 | 0.208693 | 2.71323 |
| c25077_g1_i2 | Lecithin:cholesterol acyltransferase | lipid metabolic process, O-acyltransferase activity, transferase activity, transferring acyl groups other than amino-acyl groups | 1.67584 | 0.259878 | 2.68898 |
| c26163_g1_i1 | Glycoside hydrolase, catalytic core | protein amino acid phosphorylation | 1153.48 | 192.547 | 2.58271 |
| c19524_g1_i1 | Actin-binding FH2/DRF autoregulatory | actin filament-based process, cytoskeletal protein binding | 4.53537 | 0.7919 | 2.51783 |
| c9665_g1_i1 | Photosystem I assembly protein Ycf4 | intracellular, photosynthesis, membrane | 5.54417 | 0.974385 | 2.50841 |
| c10760_g1_i1 | Short-chain dehydrogenase/reductase SDR | oxidoreductase activity | 4.10768 | 0.72274 | 2.50678 |
| c30210_g1_i1 | Natural resistance-associated macrophage protein | establishment of localization | 2.82167 | 0.516327 | 2.45019 |
| c952_g1_i1 | Glycosyl transferase, family 8 | transferase activity | 17.2211 | 3.24486 | 2.40795 |
| c19509_g1_i2 | Peptidase aspartic, catalytic | hydrolase activity, endopeptidase activity | 6.95995 | 1.3379 | 2.37911 |
| c41099_g1_i1 | Peptidase S9A, oligopeptidase, N-terminal beta-propeller | peptidase activity, acting on L-amino acid peptides, hydrolase activity | 4.30695 | 0.889805 | 2.27511 |
| c34076_g1_i1 | Lipase, class 3 | carboxylesterase activity, ipid metabolic process | 1.68515 | 0.351639 | 2.26071 |
| c21374_g1_i1 | Apoptosis regulator, Bcl-2 protein, BAG | protein binding, regulation of programmed cell death | 9.99251 | 2.13066 | 2.22955 |
| c60674_g1_i1 | Methyltransferase type 11 | metabolic process, catalytic activity | 456.776 | 97.6225 | 2.2262 |
| c1774_g1_i1 | Fringe-like | catalytic activity | 1.0361 | 0.232153 | 2.15801 |
| c26045_g2_i1 | Glycosyl transferase, family 31 | cellular carbohydrate metabolic process, glycoprotein metabolic process | 1.7144 | 0.38703 | 2.14719 |
| c12780_g1_i1 | Chloroplast Ycf2 | purine ribonucleotide binding, | 4.25313 | 0.972831 | 2.12826 |
| c19964_g2_i1 | Cytochrome f | cation binding, ion binding | 5.11177 | 1.23734 | 2.04658 |
| c23500_g1_i1 | Zinc/iron permease, fungal/plant | transporter activity | 103.071 | 25.3454 | 2.02384 |
| c19132_g1_i1 | Transferase | transferase activity, transferring acyl groups other than amino-acyl group | 20.9856 | 5.22493 | 2.00592 |
| c6097_g1_i1 | Peptidoglycan-binding lysin domain | cell wall macromolecule metabolic process, cell wall organization or biogenesis | 8.9963 | 0.0606335 | 7.21307 |
| c23932_g1_i1 | Protein kinase, catalytic domain | protein binding, purine ribonucleotide binding, ribonucleotide binding | 12.6678 | 0.236534 | 5.74297 |
| c35395_g1_i1 | Cytochrome P450, E-class, group I | iron ion binding, cation binding, ion binding | 3.61597 | 0.114082 | 4.98625 |
| c34428_g1_i1 | Glutathione S-transferase/chloride channel, C-terminal | toxin catabolic process, cellular process, secondary metabolic process | 4.21003 | 0.141787 | 4.89203 |
| c316_g1_i1 | Alcohol dehydrogenase superfamily, zinc-containing | cation binding, metabolic process | 5.07495 | 0.20188 | 4.65183 |
| c41007_g1_i1 | Calcium/calmodulin-dependent protein kinase-like | purine ribonucleotide binding, ribonucleotide binding | 36.787 | 2.08729 | 4.13949 |
| c22997_g1_i1 | DNA-binding WRKY | nitrogen compound metabolic process, regulation of nitrogen compound metabolic process | 34.955 | 3.60165 | 3.27877 |
| c27492_g1_i1 | Heat shock protein DnaJ, N-terminal | protein binding, heat shock protein binding | 2.31179 | 0.240356 | 3.26577 |
| c9426_g1_i1 | Ribosomal protein S21e | gene expression, ellular macromolecule metabolic process, cellular macromolecule biosynthetic process | 57.683 | 6.71888 | 3.10185 |
| c46789_g1_i1 | Phosphate-induced protein 1 | cell part | 7.3674 | 0.877098 | 3.07034 |
| c5953_g1_i1 | Immunoglobulin E-set |  | 1.81182 | 0.221025 | 3.03516 |
| c11295_g1_i1 | UDP-glucuronosyl/UDP-glucosyltransferase | transferase activity, transferring hexosyl groups, metabolic process | 74.8497 | 9.85568 | 2.92497 |
| c26125_g1_i1 | 2-phosphoglycolate phosphatase, eukaryotic | phosphatase activity, phosphoric ester hydrolase activity, hydrolase activity | 2.53171 | 0.352487 | 2.84447 |
| c20106_g1_i1 | Amino acid transporter, transmembrane | membrane, cell part | 49.0361 | 7.0048 | 2.80743 |
| c7067_g1_i1 | Ubiquitin supergroup | Ubiquitin supergroup | 1.13564 | 0.164058 | 2.79123 |
| c25489_g1_i1 | Pyridoxal phosphate-dependent decarboxylase | cofactor binding, vitamin B6 binding, cellular process | 4.08296 | 0.638367 | 2.67716 |
| c26755_g1_i1 | Heat shock protein Hsp20 | Stress tolerance | 72.877 | 11.916 | 2.61256 |
| c55251_g1_i1 | Polyketide cyclase/dehydrase | response to stimulus, transporter activity | 3.04438 | 0.503385 | 2.59641 |
| c4819_g1_i2 | Oxoglutarate/iron-dependent oxygenase | oxidoreductase activity, catalytic activity | 1.49045 | 0.247939 | 2.5877 |
| c6781_g1_i1 | Oligopeptide transporter OPT superfamily | cellular protein metabolic process | 1.96402 | 0.342274 | 2.52058 |
| c7487_g1_i1 | Glycosyltransferase AER61, uncharacterised | transferase activity, transferring glycosyl groups | 1.8591 | 0.327893 | 2.5033 |
| c10457_g1_i1 | Haem peroxidase, plant/fungal/bacterial | antioxidant activity, iron ion binding | 0.992566 | 0.177196 | 2.48582 |
| c26906_g1_i1 | Chalcone/stilbene synthase, C-terminal | transferase activity, transferring acyl groups other than amino-acyl groups | 15.4568 | 2.84334 | 2.44258 |
| c56072_g1_i1 | Signal transduction response regulator, receiver domain | regulation of gene expression | 2.15498 | 0.401015 | 2.42595 |
| c15574_g1_i1 | Late embryogenesis abundant protein, type 2 |  | 82.8577 | 15.5395 | 2.4147 |
| c55573_g1_i1 | Transcription factor GRAS |  | 1.57119 | 0.297421 | 2.40128 |
| c1168_g1_i1 | Thaumatin, pathogenesis-related | Thaumatin, pathogenesis-related | 1.46923 | 0.282999 | 2.37619 |
| c11391_g1_i1 | Glycoside hydrolase, family 16 | cellular polysaccharide metabolic process, cellular glucan metabolic process | 37.0488 | 7.44082 | 2.31589 |
| c48066_g1_i1 | C2 membrane targeting protein |  | 97.281 | 21.6904 | 2.1651 |
| c16971_g1_i1 | Heat shock protein 70 | adenyl nucleotide binding, nucleoside binding | 122.391 | 28.3211 | 2.11155 |
| c25783_g2_i1 | Lipase, GDSL | lipid metabolic process, O-acyltransferase activity, transferase activity, transferring acyl groups other than amino-acyl groups | 5.88588 | 1.38204 | 2.09046 |
| c24656_g1_i2 | RNA ligase/cyclic nucleotide phosphodiesterase | nitrogen compound metabolic process, cellular RNA metabolic process | 3.61589 | 0.864874 | 2.06379 |
|  | | | | | |
| **Above ambient versus Ambient: Significant DEGs down-regulated** | | | | | |
| c26287_g2_i4 | C1-like | oxidation-reduction process | 2.42595 | 9.84583 | -2.02096 |
| c424_g1_i1 | Bet v I allergen | intracellular membrane-bounded organelle, response to bacterium | 8.71489 | 36.2468 | -2.0563 |
| c20179_g1_i1 | Cyclin, C-terminal | membrane-bounded organelle | 5.71742 | 24.2026 | -2.08172 |
| c22451_g2_i1 | DVL |  | 24.3457 | 104.361 | -2.09984 |
| c21553_g3_i1 | Aux/IAA-ARF-dimerisation | developmental responses | 5.9364 | 26.933 | -2.18172 |
| c26262_g1_i1 | Auxin responsive SAUR protein |  | 30.1634 | 140.939 | -2.2242 |
| c14050_g1_i1 | Dimeric alpha-beta barrel | small molecule metabolic process | 26.4753 | 124.825 | -2.23718 |
| c14866_g1_i1 | Aldo/keto reductase | oxidoreductase activity, catalytic activity | 23.9468 | 119.923 | -2.32421 |
| c20451_g1_i1 | Plant disease resistance response protein |  | 4.81872 | 24.7507 | -2.36075 |
| c17735_g1_i1 | Haem peroxidase |  | 23.395 | 127.473 | -2.44592 |
| c19928_g1_i1 | Glyoxalase/bleomycin resistance protein/dioxygenase |  | 4.65208 | 27.8038 | -2.57933 |
| c23518_g1_i1 | Histone H5 | chromosome organization, cellular component biogenesis | 116.015 | 693.403 | -2.57939 |
| c18870_g1_i1 | RmlC-like jelly roll fold | gene expression | 0.863525 | 5.98193 | -2.7923 |
| c15907_g1_i1 | Major intrinsic protein | cellular process | 11.2891 | 89.862 | -2.99278 |
| c26534_g1_i4 | Disease resistance protein | purine ribonucleotide binding | 0.379513 | 2.19912 | -2.5347 |
| c21564_g1_i1 | Histone core | chromosome organization | 20.5736 | 117.618 | -2.51524 |
| c3941_g1_i1 | Pectinesterase inhibitor | enzyme inhibitor activity, pectinesterase activity, hydrolase activity | 2.18606 | 9.09134 | -2.05616 |
| c14091_g1_i1 | Barwin-related endoglucanase | nitrogen compound metabolic process, chitin metabolic process | 12.6426 | 60.2455 | -2.25256 |
| c12921_g1_i1 | Ribonuclease T2 | cellular amine metabolic process | 3.52548 | 17.6307 | -2.3222 |
| c24970_g1_i1 | FAS1 domain | response to organic cyclic substance | 2.81384 | 15.0378 | -2.41798 |
| c20638_g1_i1 | Pectate lyase/Amb allergen | carbon-oxygen lyase activity, carbon-oxygen lyase activity, acting on polysaccharides | 2.77812 | 17.461 | -2.65195 |
| c761_g1_i1 | Ribosomal protein S30Ae/sigma 54 modulation protein | intracellular membrane-bounded organelle | 19.1458 | 129.243 | -2.75498 |
| c40752_g1_i1 | Cupredoxin | copper ion binding | 9.27306 | 63.1562 | -2.76781 |
| c11006_g1_i1 | Heavy metal transport/detoxification protein | establishment of localization | 7.2381 | 51.6165 | -2.83415 |
