# Supplementary material for: Transcriptome analysis reveals plasticity in gene regulation due to environmental cues in Primula sikkimensis, a high altitude plant species
Source: BMC Genomics. 2019 Dec 17;20:989. doi: 10.1186/s12864-019-6354-1 (PMC6916092; doi:10.1186/s12864-019-6354-1)
Supplement: Supplementary file 9 — Additional file 9: Table S4. Pathway details. [file 12864_2019_6354_MOESM9_ESM.docx]

**Table S4** – **A:** Pathway details of Below ambient site.

| #Term | Database | ID | Input number | Background number | P-Value | Corrected P-Value | Input |
| --- | --- | --- | --- | --- | --- | --- | --- |
| Biosynthesis of secondary metabolites | KEGG PATHWAY | ath01110 | 17 | 1076 | 1.20E-06 | 0.000187 | c25423_g2_i1\|c14597_g1_i1\|c13836_g1_i1\|c19444_g1_i1\|c20496_g1_i1\|c25489_g1_i1\|c15061_g1_i1\|c22171_g1_i1\|c47005_g1_i1\|c26519_g2_i1\|c12925_g1_i1\|c22669_g1_i1\|c42549_g1_i1\|c28257_g1_i1\|c17568_g1_i1\|c23887_g1_i1\|c25059_g1_i4 |
| Flavonoid biosynthesis | KEGG PATHWAY | ath00941 | 4 | 21 | 2.64E-06 | 0.00036 | c15061_g1_i1\|c22669_g1_i1\|c22171_g1_i1\|c47005_g1_i1 |
| Metabolic pathways | KEGG PATHWAY | ath01100 | 20 | 1910 | 5.47E-05 | 0.00351 | c25423_g2_i1\|c14597_g1_i1\|c13836_g1_i1\|c42549_g1_i1\|c23174_g1_i1\|c20496_g1_i1\|c25489_g1_i1\|c15061_g1_i1\|c21020_g1_i3\|c22171_g1_i1\|c8824_g1_i1\|c26519_g2_i1\|c12925_g1_i1\|c22669_g1_i1\|c47005_g1_i1\|c28257_g1_i1\|c17568_g1_i1\|c23887_g1_i1\|c57533_g1_i1\|c25059_g1_i4 |
| Phenylalanine metabolism | KEGG PATHWAY | ath00360 | 2 | 42 | 0.01292 | 0.135532 | c23887_g1_i1\|c42549_g1_i1 |
| Phenylpropanoid biosynthesis | KEGG PATHWAY | ath00940 | 3 | 157 | 0.025137 | 0.18434 | c23887_g1_i1\|c13836_g1_i1\|c42549_g1_i1 |
| Oxidative phosphorylation | KEGG PATHWAY | ath00190 | 3 | 162 | 0.027203 | 0.18434 | c21020_g1_i3\|c25059_g1_i4\|c16979_g1_i1 |
| Pentose and glucuronate interconversions | KEGG PATHWAY | ath00040 | 2 | 81 | 0.042126 | 0.212472 | c57533_g1_i1\|c13691_g1_i1 |
| Phagosome | KEGG PATHWAY | ath04145 | 2 | 86 | 0.046798 | 0.213625 | c21020_g1_i3\|c22486_g1_i1 |
| Protein processing in endoplasmic reticulum | KEGG PATHWAY | ath04141 | 3 | 212 | 0.052633 | 0.224393 | c16121_g1_i1\|c26655_g1_i2\|c23820_g1_i5 |
| Selenocompound metabolism | KEGG PATHWAY | ath00450 | 1 | 18 | 0.07188 | 0.242789 | c28257_g1_i1 |
| Cysteine and methionine metabolism | KEGG PATHWAY | ath00270 | 2 | 112 | 0.073807 | 0.246248 | c12925_g1_i1\|c28257_g1_i1 |
| Biosynthesis of amino acids | KEGG PATHWAY | ath01230 | 3 | 255 | 0.081029 | 0.253147 | c12925_g1_i1\|c28257_g1_i1\|c26519_g2_i1 |
| Photosynthesis - antenna proteins | KEGG PATHWAY | ath00196 | 1 | 22 | 0.086347 | 0.255593 | c8824_g1_i1 |
| Isoquinoline alkaloid biosynthesis | KEGG PATHWAY | ath00950 | 1 | 23 | 0.089929 | 0.256168 | c25489_g1_i1 |
| Plant hormone signal transduction | KEGG PATHWAY | ath04075 | 3 | 271 | 0.092982 | 0.257038 | c22197_g1_i2\|c20050_g1_i1\|c55251_g1_i1 |
| Amino sugar and nucleotide sugar metabolism | KEGG PATHWAY | ath00520 | 2 | 135 | 0.100856 | 0.260745 | c57533_g1_i1\|c14384_g1_i1 |
| Cutin, suberine and wax biosynthesis | KEGG PATHWAY | ath00073 | 1 | 28 | 0.10763 | 0.267947 | c23174_g1_i1 |
| Carotenoid biosynthesis | KEGG PATHWAY | ath00906 | 1 | 29 | 0.111129 | 0.268829 | c25423_g2_i1 |
| Ubiquinone and other terpenoid-quinone biosynthesis | KEGG PATHWAY | ath00130 | 1 | 35 | 0.13184 | 0.292353 | c23887_g1_i1 |
| Fatty acid elongation | KEGG PATHWAY | ath00062 | 1 | 35 | 0.13184 | 0.292353 | c19444_g1_i1 |
| Circadian rhythm - plant | KEGG PATHWAY | ath04712 | 1 | 36 | 0.135245 | 0.293117 | c47005_g1_i1 |
| Tyrosine metabolism | KEGG PATHWAY | ath00350 | 1 | 40 | 0.148734 | 0.313259 | c25489_g1_i1 |
| Ascorbate and aldarate metabolism | KEGG PATHWAY | ath00053 | 1 | 41 | 0.152073 | 0.318448 | c57533_g1_i1 |
| Alanine, aspartate and glutamate metabolism | KEGG PATHWAY | ath00250 | 1 | 48 | 0.175088 | 0.341109 | c20496_g1_i1 |
| Phenylalanine, tyrosine and tryptophan biosynthesis | KEGG PATHWAY | ath00400 | 1 | 57 | 0.203772 | 0.360316 | c26519_g2_i1 |
| Terpenoid backbone biosynthesis | KEGG PATHWAY | ath00900 | 1 | 58 | 0.206897 | 0.363486 | c14597_g1_i1 |
| Citrate cycle (TCA cycle) | KEGG PATHWAY | ath00020 | 1 | 63 | 0.222343 | 0.375504 | c25059_g1_i4 |
| Glutathione metabolism | KEGG PATHWAY | ath00480 | 1 | 93 | 0.308953 | 0.45922 | c23102_g1_i1 |
| RNA degradation | KEGG PATHWAY | ath03018 | 1 | 112 | 0.358789 | 0.507703 | c16229_g1_i1 |
| Endocytosis | KEGG PATHWAY | ath04144 | 1 | 142 | 0.430323 | 0.570453 | c26655_g1_i2 |
| Plant-pathogen interaction | KEGG PATHWAY | ath04626 | 1 | 167 | 0.483851 | 0.615964 | c16121_g1_i1 |
| Spliceosome | KEGG PATHWAY | ath03040 | 1 | 192 | 0.532391 | 0.661548 | c26655_g1_i2 |
| Starch and sucrose metabolism | KEGG PATHWAY | ath00500 | 1 | 202 | 0.550516 | 0.671076 | c57533_g1_i1 |
| Carbon metabolism | KEGG PATHWAY | ath01200 | 1 | 262 | 0.645534 | 0.740222 | c25059_g1_i4 |
|  | | | | | | | |
| **B:** Pathway details of Above ambient site. | | | | | | | |
| #Term | Database | ID | Input number | Background number | P-Value | Corrected P-Value | Input |
| Photosynthesis | KEGG PATHWAY | ath00195 | 3 | 77 | 0.001962 | 0.045133 | c19964_g2_i1\|c25075_g2_i3\|c9665_g1_i1 |
| Plant hormone signal transduction | KEGG PATHWAY | ath04075 | 4 | 271 | 0.010458 | 0.112921 | c9465_g1_i1\|c18341_g1_i1\|c55573_g1_i1\|c26262_g1_i1 |
| Metabolic pathways | KEGG PATHWAY | ath01100 | 12 | 1910 | 0.014729 | 0.112921 | c14171_g1_i1\|c16611_g1_i1\|c25075_g2_i3\|c20889_g1_i1\|c9665_g1_i1\|c19964_g2_i1\|c60674_g1_i1\|c15815_g1_i1\|c23617_g6_i2\|c26510_g9_i1\|c25489_g1_i1\|c12479_g1_i1 |
| Biosynthesis of secondary metabolites | KEGG PATHWAY | ath01110 | 7 | 1076 | 0.049853 | 0.180783 | c14171_g1_i1\|c16611_g1_i1\|c26510_g9_i1\|c60674_g1_i1\|c15815_g1_i1\|c25489_g1_i1\|c12479_g1_i1 |
| Pyrimidine metabolism | KEGG PATHWAY | ath00240 | 2 | 116 | 0.051592 | 0.180783 | c20889_g1_i1\|c26510_g9_i1 |
| Flavonoid biosynthesis | KEGG PATHWAY | ath00941 | 1 | 21 | 0.065749 | 0.180783 | c12479_g1_i1 |
| Isoquinoline alkaloid biosynthesis | KEGG PATHWAY | ath00950 | 1 | 23 | 0.07151 | 0.180783 | c25489_g1_i1 |
| Endocytosis | KEGG PATHWAY | ath04144 | 2 | 142 | 0.073145 | 0.180783 | c19568_g1_i1\|c19983_g2_i1 |
| Phenylpropanoid biosynthesis | KEGG PATHWAY | ath00940 | 2 | 157 | 0.086677 | 0.180783 | c15815_g1_i1\|c14171_g1_i1 |
| Purine metabolism | KEGG PATHWAY | ath00230 | 2 | 158 | 0.087604 | 0.180783 | c20889_g1_i1\|c26510_g9_i1 |
| Ubiquinone and other terpenoid-quinone biosynthesis | KEGG PATHWAY | ath00130 | 1 | 35 | 0.105345 | 0.180783 | c60674_g1_i1 |
| Circadian rhythm - plant | KEGG PATHWAY | ath04712 | 1 | 36 | 0.108109 | 0.180783 | c12479_g1_i1 |
| Tyrosine metabolism | KEGG PATHWAY | ath00350 | 1 | 40 | 0.119081 | 0.180783 | c25489_g1_i1 |
| Nitrogen metabolism | KEGG PATHWAY | ath00910 | 1 | 42 | 0.124517 | 0.180783 | c11933_g1_i1 |
| Limonene and pinene degradation | KEGG PATHWAY | ath00903 | 1 | 44 | 0.129919 | 0.180783 | c16611_g1_i1 |
| RNA polymerase | KEGG PATHWAY | ath03020 | 1 | 45 | 0.132608 | 0.180783 | c20889_g1_i1 |
| Stilbenoid, diarylheptanoid and gingerol biosynthesis | KEGG PATHWAY | ath00945 | 1 | 46 | 0.135289 | 0.180783 | c16611_g1_i1 |
| Protein processing in endoplasmic reticulum | KEGG PATHWAY | ath04141 | 2 | 212 | 0.141482 | 0.180783 | c26755_g1_i1\|c19983_g2_i1 |
| Pentose and glucuronate interconversions | KEGG PATHWAY | ath00040 | 1 | 81 | 0.224129 | 0.271314 | c12041_g1_i1 |
| Glutathione metabolism | KEGG PATHWAY | ath00480 | 1 | 93 | 0.252461 | 0.29033 | c34428_g1_i1 |
| Oxidative phosphorylation | KEGG PATHWAY | ath00190 | 1 | 162 | 0.396608 | 0.424366 | c23617_g6_i2 |
| Plant-pathogen interaction | KEGG PATHWAY | ath04626 | 1 | 167 | 0.405915 | 0.424366 | c19077_g1_i1 |
| Spliceosome | KEGG PATHWAY | ath03040 | 1 | 192 | 0.450362 | 0.450362 | c19983_g2_i1 |
